# Establishment of a Cre/loxP recombination system for N-terminal epitope tagging of genes in *Tetrahymena*

**DOI:** 10.1186/1471-2180-10-191

**Published:** 2010-07-13

**Authors:** Clara Jana-Lui Busch, Alexander Vogt, Kazufumi Mochizuki

**Affiliations:** 1 Institute of Molecular Biotechnology of the Austrian Academy of Sciences (IMBA), Dr. Bohr-Gasse 3, A-1030 Vienna, Austria

## Abstract

**Background:**

Epitope tagging is a powerful strategy to study the function of proteins. Although tools for C-terminal protein tagging in the ciliated protozoan *Tetrahymena thermophila *have been developed, N-terminal protein tagging in this organism is still technically demanding.

**Results:**

In this study, we have established a Cre/loxP recombination system in *Tetrahymena *and have applied this system for the N-terminal epitope tagging of *Tetrahymena *genes. Cre can be expressed in *Tetrahymena *and localizes to the macronucleus where it induces precise recombination at two loxP sequences in direct orientation in the *Tetrahymena *macronuclear chromosome. This Cre/loxP recombination can be used to remove a loxP-flanked drug-resistance marker from an N-terminal tagging construct after it is integrated into the macronucleus.

**Conclusions:**

The system established in this study allows us to express an N-terminal epitope tagged gene from its own endogenous promoter in *Tetrahymena*.

## Background

Epitope tagging has been widely used for the analysis of protein localization, interaction, and function (reviewed in [1]). It is extremely useful in studying the proteins of the ciliated protozoan *Tetrahymena thermophila *because epitope tags can be introduced efficiently into endogenous chromosomal loci by homologous recombination in this organism [2].

In many cases, a protein of interest is tagged by introducing a tag at its C-terminus [3-5] because a drug-resistance marker, which must be introduced in proximity to the tag for the establishment of transgenic strains, rarely disturbs the gene promoter if it is inserted downstream of a target gene; thus, the tagged protein can be expressed at its endogenous levels. We previously established a set of convenient modules designed for PCR- and plasmid-based C-terminal tagging (Kataoka et al. submitted). However, sometimes a C-terminal tag renders the protein dysfunctional, disturbs the localization of the protein, or interferes with the protein's interaction with other molecules. In these cases, tagging the protein at its N-terminus might be advised.

There is a drawback to the N-terminal epitope tagging strategy in general: an insertion of a drug-resistance marker into the upstream region of a gene could disturb its promoter activity. This possibility is especially an issue in the *Tetrahymena *system because intergenic sequences are relatively short in this organism [6]. To avoid this problem, in previous experiments, N-terminally tagged proteins were expressed from ectopic genome locations, such as rDNA or β-tubulin 1 (*BTU1*) loci, and/or by ectopic promoters at their endogenous loci [7-10]. However, expression levels and patterns of these ectopically expressed N-terminally tagged proteins could differ from those of their endogenous counterparts and thus might cause mislocalization of proteins or artificial interaction with other molecules. Alternatively, a drug-resistance marker can be inserted into the downstream region of a gene for N-terminal tagging. However, in this case, the entire coding sequence and both the upstream and the downstream flanking sequences of the gene have to be cloned as a single construct, which is sometimes not easy for large genes. In addition, if homologous recombination occurs within the coding sequence, an epitope tag at the N-terminus in the construct would be lost. Moreover, the inserted selectable marker could disturb the expression of the downstream gene.

We intended to use the Cre/loxP recombination system to solve these problems. Cre is a recombinase from the bacteriophage P1 that mediates intramolecular and intermolecular site-specific recombination between two loxP sites [11]. A loxP site consists of two 13 bp inverted repeats separated by an 8 bp asymmetric spacer region. Two loxP sites in direct orientation dictate excision of the intervening DNA between the sites leaving one loxP site behind. This precise excision of DNA can remove a loxP-flanked drug-resistance marker from the N-terminal tagging construct after it is integrated into the macronucleus, and thus allows us to introduce epitope tags to the N-terminus of a gene of interest without disturbing its promoter. Here, we describe the establishment of a Cre/loxP recombination system in *Tetrahymena *and demonstrate its usefulness for the N-terminal tagging of *Tetrahymena *genes.

## Results

### Cre-recombinase localizes to the macronucleus in *Tetrahymena*

**Figure 1:**
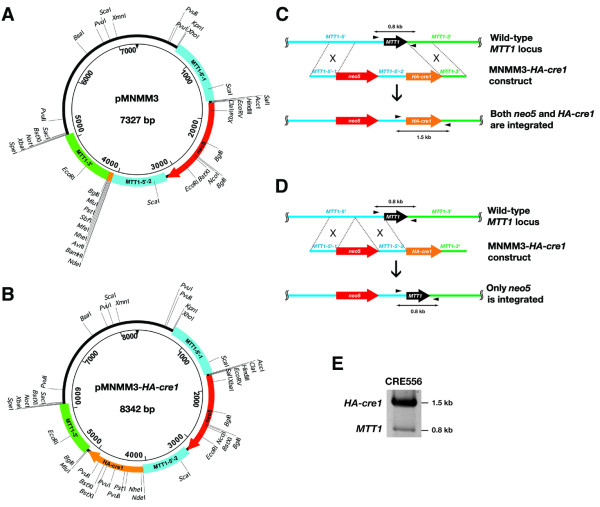
**Construction of a Cre-recombinase expressing *Tetrahymena *strain**. (A, B) Plasmid maps of pMNMM3 (A) and pMNMM3-*HA-cre1 *(B). (C, D) Two possible homologous recombination events between the MNMM3-HA-cre1 construct and the *Tetrahymena MTT1 *genomic locus. Homologous recombination at "*MTT1*-5'(1)" and "*MTT1*-3'" integrates both *neo5 *and the *HA-cre1 *gene (C), whereas recombination at "*MTT1*-5'(1)" and "*MTT1*-5'(2)" integrates only the *neo5 *cassette into the genome (D). (E) PCR analysis of the CRE556 strain. Genomic DNA from the CRE556 strain was used to amplify the *HA-cre1*-containing locus (*HA-cre1*) and wild-type *MTT1 *locus (*MTT1*). The positions of the primers are represented by arrowheads in (C).

To test if Cre-recombinase can be expressed in *Tetrahymena*, we designed an inducible expression system for Cre. First, we constructed an expression cassette (pMNMM3, Fig. 1A) by which we can replace the endogenous *MTT1 *coding sequence with any gene of interest. In this cassette, genes can be expressed under the control of the *MTT1 *promoter, which is induced by the presence of heavy metals such as cadmium [12]. We synthesized a Cre-encoding gene, *cre1*, in which the codon-usage was optimized for *Tetrahymena*. An HA-tag was added to the N-terminus of *cre1 *and the construct was inserted into pMNMM3 to produce pMNMM3-*HA-cre1 *(Fig. 1B). Finally, the expression construct was excised from the vector backbone of pMNMM3-*HA-cre1 *and introduced into the macronucleus of the *Tetrahymena *B2086 strain by homologous recombination (Fig. 1C). Cells possessing the Cre-expression construct were selected by their resistance against paromomycin because the construct contains a *neo5 *cassette, which confers resistance to this drug in *Tetrahymena *cells. The *neo5 *cassette has a similar structure as *neo2 *(Gaertig et al. 1994) but has a codon-optimized neomycin-resistance gene (*neoTet*, [13]) instead of the bacteriophage-derived *neo *gene.

The macronucleus is polyploid and its chromosomes randomly segregate to the daughter nuclei. By stepwise selection with increasing amounts of paromomycin, we intended to obtain strains in which the majority of the endogenous *MTT1 *loci were replaced by the Cre-expression construct. We realized that some strains became resistant to a much higher concentration of paromomycin (> 4 mg/mL) than other strains (~1 mg/mL). PCR analysis revealed that the former strains did not receive the Cre gene, probably because homologous recombination had occurred at "*MTT1*-5'-1" and "*MTT1*-5'-2" (Fig. 1D). In contrast, the latter strains contained both *neo5 *and the *HA-cre1 *gene, indicating that homologous recombination had occurred at "*MTT1*-5'-1" and "*MTT1*-3'"(Fig. 1C). The reason for the limited growth of HA-Cre1p-expressing cells is probably due to weak *MTT1 *promoter activity caused by a paromomycin-induced stress. HA-Cre1p expression suppresses cell growth (see below), which might be the reason for the limited resistance of the HA-Cre1p-expressing strain to higher concentrations of paromomycin. We used one of the latter *HA-cre1 *possessing strains, CRE556, for further study. In this strain, most of the endogenous *MTT1 *loci were replaced with the *HA-cre1 *expression construct (Fig. 1E).

**Figure 2:**
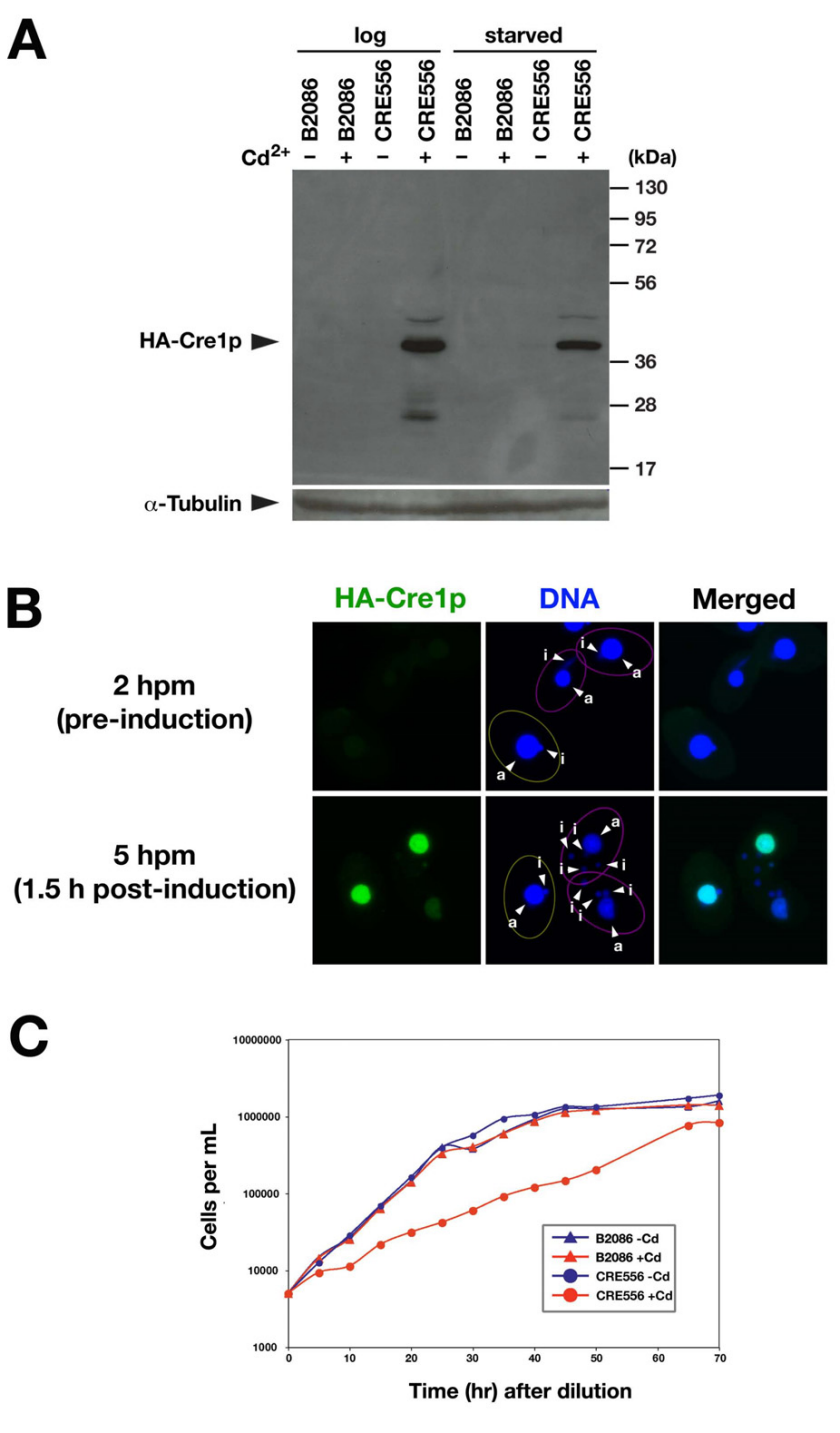
**Expression of Cre-recombinase in *Tetrahymena***. (A) Expression of HA-Cre1p in the CRE556 strain is induced by the presence of cadmium ions. B2086 (wild-type) and CRE556 cells were cultured in the nutrient-rich 1× SPP medium (log) or in 10 mM Tris (pH 7.5) (starved) and were treated with (+) or without (-) CdCl_2_. For log and starved cells, 1 μg/mL and 50 ng/mL CdCl_2 _were used, respectively. HA-Cre1p was detected by western blotting using an anti-HA antibody. For the loading control, the membrane was stripped using a 2-mercaptoethanol- and SDS-containing buffer and re-probed with antibody against α-tubulin. (B) HA-Cre1p localizes to the macronucleus in *Tetrahymena*. CRE556 was mated with a wild-type strain and HA-Cre1p expression was induced at 3.5 hr post-mixing (hpm) by adding 50 ng/mL CdCl_2_. Cells were fixed at 2 hpm (before induction) or at 5 hpm (1.5 hr after induction) and HA-Cre1p was localized using an anti-HA antibody. DNA was counter-stained by DAPI. In each picture, a non-mating (starved) cell (circled with yellow line) and a pair of mating cells (circled with magenta line) are shown. The macro- and micronuclei are marked with "a" and "i", respectively. (C) Expression of HA-Cre1p suppresses growth of *Tetrahymena*. B2086 (wild-type) or CRE556 were diluted to 5,000 cells/mL with 1× SPP medium with or without 1 μg/mL CdCl_2_. At indicated time after dilution, cells were counted to monitor cell growth.

To ask if HA-Cre1p can be expressed in *Tetrahymena *cells, the CRE556 strain was cultured either in a nutrient-rich (Super Proteose Peptone (SPP)) medium with or without 1 μg/ml CdCl_2 _or in 10 mM Tris (pH 7.5) with or without 50 ng/ml CdCl_2 _and HA-Cre1p expression was detected by western blotting using an anti-HA antibody. As shown in Fig. 2A, a ~40 kDa band, which corresponds to the predicted molecular weight of HA-Cre1p (39.7 kDa), was detected only when the CRE556 strain was treated with CdCl_2_. Therefore, the CRE556 strain can express HA-Cre1p in a CdCl_2_-dependent manner. 1 μg/ml CdCl_2 _in SPP medium and 50 ng/ml CdCl_2 _in 10 mM Tris induced a similar expression level of HA-Cre1p. This is consistent with the fact that the *MTT1 *promoter is activated at lower concentration in cells starved in 10 mM Tris than in those growing in SPP medium [12].

Immunofluorescence staining using an anti-HA antibody indicated that HA-Cre1p localized to the macronucleus both in the vegetative cells and conjugating cells (Fig. 2B) after its induction by CdCl_2_. Importantly, when the CRE556 strain was crossed with a wild-type strain, HA-Cre1p protein was detected in both cells of a pair (Fig. 2B). This result indicates that either HA-Cre1p protein or HA-Cre1p mRNA can be transferred from the CRE556 strain to the partner cell during conjugation. This is not surprising because it is known that RNA and protein is exchanged between mating pairs [14]. Therefore, the CRE556 strain could be used to induce homologous recombination at loxP sites introduced into the macronucleus of any cell that can mate with this strain.

### Expression of Cre-recombinase suppresses the growth of *Tetrahymena*

Because Cre is a nuclease, its expression might be genotoxic to *Tetrahymena *cells. We tested this possibility by analyzing the growth of the CRE556 strain with and without induction of HA-Cre1p expression. Indeed, growth of the CRE556 strain was significantly suppressed when the cells were cultured in the presence of 1 μg/mL CdCl_2_, whereas the same amount of CdCl_2 _had little effect on the growth of the wild-type strain (Fig. 2C). The growth defect in the CRE556 strain is not due to a reduced copy number of the *MTT1 *gene as expression of *HA-cre1* from the *BTU1 *locus (Supplementary Fig. S1 in Additional file 1) caused similar growth suppression in the presence of CdCl_2 _(Fig. 2D). These results indicate that the expression of HA-Cre1p has a negative, possibly genotoxic effect on the growth of *Tetrahymena *cells. Therefore, it is necessary to minimize the exposure of cells to Cre1p when it is used for *Tetrahymena *transgenesis. The inducible Cre expression system aids in minimizing this toxic effect.

### Cre-recombinase can induce precise recombination at loxP sites

**Figure 3:**
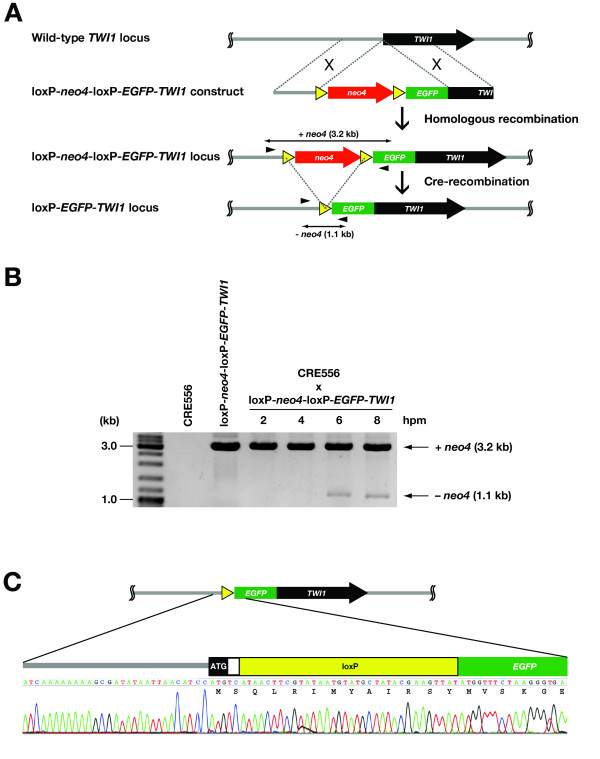
**Cre-recombinase induces precise recombination at loxP sites**. (A) Diagrams of the wild-type *TWI1*, loxP-*neo4*-loxP-*EGFP*-*TWI1 *and loxP-*EGFP*-*TWI1 *loci. The loxP-*neo4*-loxP-*EGFP*-*TWI1 *construct was introduced to the *TWI1 *locus by homologous recombination. The *neo4 *cassette was removed from the loxP-*neo4*-loxP-*EGFP*-*TWI1 *locus by Cre-mediated recombination to produce the loxP-*EGFP*-*TWI1 *locus. The arrowheads represent the primers used for the DNA excision analysis shown in Fig. 3B and Fig. 4B. (B) Cre-induced recombination at loxP-*neo4*-loxP-*EGFP*-*TWI1 *locus. Total genomic DNA was extracted from starved CRE556 or loxP-*neo4*-loxP-*EGFP-TWI1 *cells, or mating CRE556 and loxP-*neo4*-loxP-*EGFP-TWI1 *PCR cells at 2, 4, 6 and 8 hr post-mixing (hpm) and PCR-amplified using the primers shown in Fig. 3A. The products corresponding to the non-excised loxP-*neo4*-loxP-*EGFP*-*TWI1 *locus (+*neo4*) and the excised loxP-*EGFP-TWI1 *locus (-*neo4*) are marked by arrows. (C) Sequence analysis of the loxP-*EGFP-TWI1 *locus. DNA sequence of the 1.1 kb PCR product from mating CRE556 and loxP-*neo4*-loxP-*EGFP-TWI1 *PCR cells at 8 hpm was analyzed.

To test if expression of the Cre-recombinase can induce homologous recombination at two loxP sites, we constructed a strain, loxP-*neo4*-loxP-*EGFP-TWI1*, in which the *neo4 *cassette was flanked by two loxP sequences in the *TWI1 *locus (Fig. 3A). CRE556 cells starved in 10 mM Tris (pH 7.5) were pre-treated with 50 ng/mL CdCl_2 _for 1.5 hr to induce the expression of HA-Cre1p and mated with a loxP-*neo4*-loxP-*EGFP-TWI1 *strain in 10 mM Tris (pH 7.5). Then, excision of the *neo4 *cassette was observed by PCR using the primers indicated in Fig. 3A. As shown in Fig. 3B, in addition to a ~3 kb product that corresponds to the intact loxP-*neo4*-loxP-*EGFP-TWI1 *locus, a ~1 kb PCR product that corresponds to the locus lacking *neo4 *was detected from 6 hr post-mixing (hpm) onwards. This *neo4*-excised locus will be referred to as loxP-*EGFP-TWI1*. DNA sequencing of the shorter PCR product confirmed that this product resulted from the precise excision of *neo4 *by homologous recombination of two loxP sites (Fig. 3C).

### The Cre/loxP system can be used for N-terminal epitope tagging

In the loxP-*neo4*-loxP-*EGFP-TWI1 *locus, the loxP-*neo4*-loxP sequence is inserted directly before the first methionine-coding codon of the *EGFP-TWI1 *fusion gene. Therefore, *EGFP-TWI1 *can be expressed only after the excision of the *neo4 *cassette by HA-Cre1p. This system allows us to express N-terminal EGFP-tagged Twi1p from the endogenous *TWI1 *promoter. Because the parental macronucleus is eventually destroyed at the end of conjugation, the loxP-*neo4*-loxP-*EGFP-TWI1 *locus or the *neo4*-excised loxP-*EGFP*-*TWI1 *locus is lost in the sexual progeny. Therefore, to use the loxP-*EGFP-TWI1 *locus for analyses of EGFP-Twi1p, parental cells must be recovered after the induction of conjugation between the CRE556 and the loxP-*neo4*-loxP-*EGFP-TWI1 *strains. Around a quarter of mating wild-type *Tetrahymena *cells aborts conjugation before producing zygotic nuclei and haploid meiotic micronuclear products are endoreplicated to regenerate a diploid micronucleus. Parental macronuclei are preserved in this process [15].

**Figure 4:**
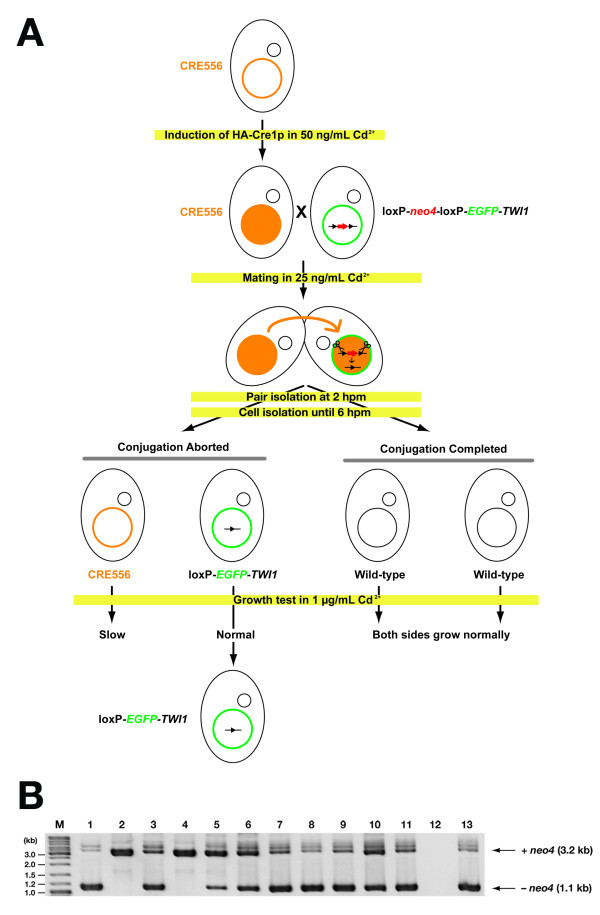
**N-terminal EGFP-tagging of *TWI1 *using Cre/loxP system**. (A) A scheme of induction of Cre-mediated loxP recombination and selection of loxP-*EGFP-TWI1 *cells. See text for details. (B) loxP excision analysis by PCR. Total genomic DNA was extracted from 13 presumptive loxP-*EGFP-TWI1 *strains and analyzed by PCR using the primers shown in Fig. 3A. The products corresponding to the non-excised loxP-*neo4*-loxP-*EGFP*-*TWI1 *locus (+*neo4*) and the excised loxP-*EGFP-TWI1 *locus (-*neo4*) are marked by arrows.

We established a method to efficiently recover cells after aborting conjugation and to distinguish the loxP-*neo4*-loxP-*EGFP-TWI1 *(or *neo4*-excised loxP-*EGFP-TWI1*) strain from CRE556. The method is schematically shown in Fig. 4A. First, individual mating pairs were isolated into drops of 1× SPP at 2 hpm and cells aborting conjugation in these drops by 6 hpm were isolated into drops of fresh 1× SPP. Because mating continues until ~10 hpm if the zygotic nuclei are successfully produced, cells separated by 6 hpm most probably maintain the parental macronuclei. Out of 64 pairs isolated we retrieved 19 sets of clones in which both sides of the separated cells continued to grow. They were then cultured in 1× SPP containing 1 μg/mL CdCl_2_. Because the expression of HA-Cre1p severely inhibits the growth of *Tetrahymena*, one side of the clones in each set was expected to grow slowly in the presence of CdCl_2_. Indeed, in 13 out of the 19 sets of the clones studied, severe growth suppression was detected in one side of the clones. In the other 6 sets, both sides of cells grew at equal speed. These are likely to represent progeny cells and were not analyzed further.

We expected that the clones growing poorly in the presence of CdCl_2 _were derived from the CRE556 strain, while the normally growing clones originated from the loxP-*neo4*-loxP-*EGFP-TWI1 *strain. Genomic DNA was extracted from the latter clones and excision of the *neo4 *cassette was observed by PCR. As shown in Fig. 4B, the PCR product corresponding to the *neo4*-excised loxP-*EGFP-TWI1 *locus was observed in 10 out of 13 clones studied. This result indicated that they indeed were derived from the loxP-*neo4*-loxP-*EGFP-TWI1 *strain and Cre-recombinase expressed in the CRE556 side of the pair was transported to the loxP-*neo4*-loxP-*EGFP-TWI1 *side and efficiently induced *neo4 *excision. Only one clone failed to produce any PCR products. This clone could be either derived from the CRE556 strain or from progeny cells that we could not correctly identify by the growth assay in the presence of CdCl_2_. Therefore, the method we established here can efficiently identify parental cells derived from a loxP-possessing strain.

**Figure 5:**
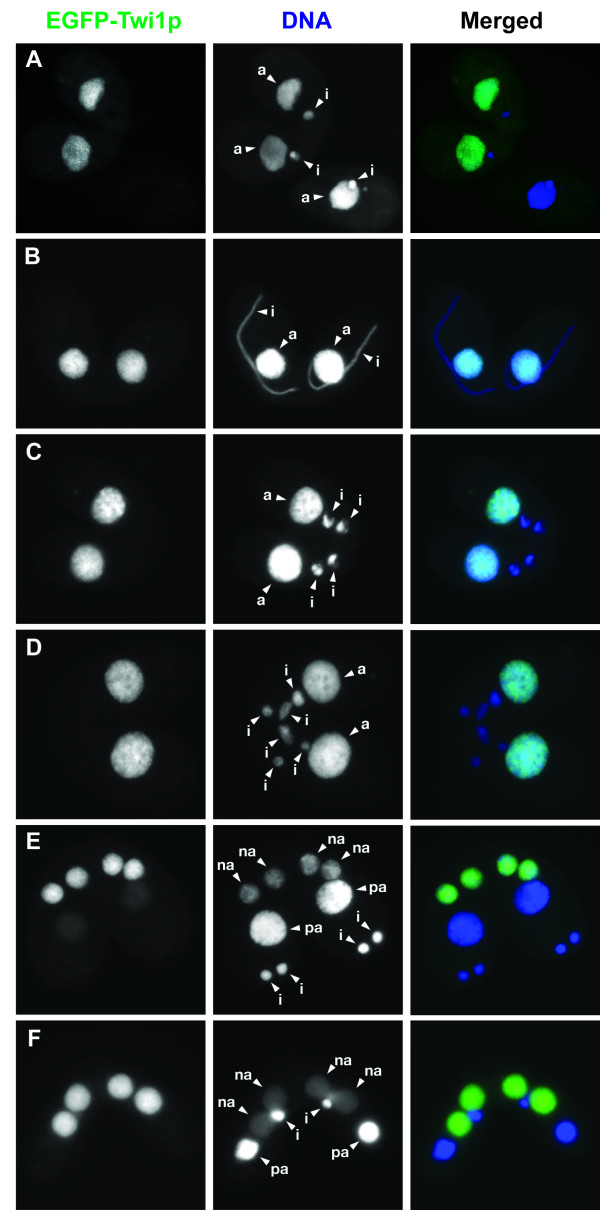
**Localization of EGFP-Twi1p**. The loxP-*EGFP-TWI1 *strain #1 (Fig. 4B) was mated with the wild-type B2086 and localization of EGFP-Twi1p at conjugation stages E1 (A, B), E2 (C), M1 (D) or L1 (E, F) was observed using fluorescence microscopy. A detailed illustration of conjugation stages can be found in [3]. DNA was counterstained by DAPI. a: macronucleus, i: micronucleus, na: new macronucleus, pa: parental macronucleus.

To assess the correct excision of the *neo4 *cassette in these clones, they were crossed with the wild-type strain CU428 and EGFP-Twi1p expression was observed. In all 3 clones (#1, #11 and #13 in Fig. 4B) analyzed, EGFP-Twi1p was exclusively expressed during conjugation and was localized to the macronucleus. EGFP-Twi1p localization of clone #1 is shown in Fig. 5. The expression of EGFP indicated that *neo4 *was most likely excised precisely at the two loxP sites because imprecise excision might cause a frame-shift that abolishes EGFP-Twi1p expression. The conjugation-specific expression and the macronuclear localization of EGFP-Twi1p mirror the properties of Twi1p in wild-type cells [3], strongly suggesting that the endogenous *TWI1 *promoter correctly drives the downstream *EGFP-TWI1 *expression.

## Discussion

In this study, we have established a Cre/loxP recombination system in *Tetrahymena *and have demonstrated that this system is useful for N-terminal EGFP tagging of the *TWI1 *gene. Although we have tested only N-terminal EGFP tagging here, we expect that this system can be applied to any type of epitope tag. However, because one loxP sequence remains after the Cre-mediated recombination event in this system, functionalities (e.g., antigenicities) of each epitope tag could be disturbed by the presence of the short peptides (SQLRIMYAIRSY, see also Fig. 3C) encoded by the loxP sequence. Therefore, validity of this system must be carefully examined for each epitope tag.

We also believe that the system established in this study can be used for internal epitope tagging. In addition, it may be safer to use this system for C-terminal epitope tagging because intergenic sequences are relatively short in *Tetrahymena *(Eisen et al. 2006) and the presence of a drug-resistance marker at the 3'-flanking region of some genes could disturb the promoter function of a neighboring gene. Moreover, similar to the "brainbow" mouse [16], combinatory use of multiple loxP mutant sequences may allow us to produce *Tetrahymena *cells expressing a protein tagged with several different epitope tags by a single transformation experiment followed by Cre-mediated recombination.

Cre/loxP recombination systems have also been used for conditional gene knockouts [17] and recycling drug-resistance markers for multiple transformations [18-20] in other model organisms. We expect that the system described here can be used for these applications in *Tetrahymena *as well. However, because *Tetrahymena *has a polyploid (~50 copies) macronucleus and because the loxP excision did not occur in all of the macronuclear copies in the condition we tested (see Fig. 4B), it will be necessary to improve the recombination efficiency to use the Cre/loxP system for these applications in *Tetrahymena*. Nonetheless, the existing technique is already applicable to recycle a drug-resistance marker. The macronuclear chromosomes segregate randomly to daughter nuclei, and thus we can obtain cells in which all copies of a locus have a loxP-excised form by phenotypic assortment [21].

We chose a relatively complex procedure to introduce Cre1p into cells: *HA-cre1* expressing cells were mated with cells possessing the loxP target locus. Because of the following two reasons, we believe this strategy has great advantage over other possible methods such as introducing the *HA-cre1* gene and a loxP target locus into the same cell. First, as shown in Fig. 2C, expression of HA-Cre1p suppresses cell growth, suggesting that HA-Cre1p is probably toxic to *Tetrahymena*. Therefore, it is necessary to minimize the exposure of cells to HA-Cre1p. Although the *MTT1* promoter, which is driving the HA-Cre1p expression, is inducible by the presence of cadmium, it is known that in the absence of cadmium leaky expression occurs [12]. In our strategy the exposure of cells to HA-Cre1p can be tightly controlled and thus potential cell damages are avoided. Second, if we introduce *HA-cre1* and a loxP-flanked marker locus into the same cell, leaky expression of HA-Cre1p from the *MTT1* promoter may eliminate the marker. This possibly makes phenotypic assortment difficult. Our strategy is free from this problem because strains containing *HA-cre1* and the loxP-flanked marker are prepared separately.

## Conclusions

Cre-recombinase can be expressed in *Tetrahymena *and localizes to the macronucleus. It can induce precise recombination at two loxP sequences in direct orientation in the macronuclear chromosome. The successful construction of a strain expressing N-terminal EGFP-tagged *TWI1 *from the endogenous *TWI1 *locus let us conclude that the Cre/loxP recombination system established in this study is a useful tool for N-terminal epitope tagging of *Tetrahymena *genes.

## Methods

### *Tetrahymena *strains and culture conditions

Wild-type B2086, CU427 and CU428 strains of *Tetrahymena thermophila *were provided by Dr. P. J. Bruns (Cornell University). These strains are also available at the *Tetrahymena *Stock Center (Cornell University). The cells were grown at 30°C in 1× Super Proteose Peptone (SPP) medium [22] containing 2% proteose peptone. Before mating, the cells were washed and resuspended (~5 × 10^5 ^cells/mL) in 10 mM Tris buffer (pH 7.5). After 12-24 h of incubation at 30°C, equal numbers of cells were mixed and mated.

### Oligo DNAs

Oligo DNAs used in this study are listed in Table 1.

**Table 1:** Oligo DNAs used in this study

**Names**	**DNA Sequences (5' to 3')**
MTT5'FWXho	GGCTCGAGTCTTTGCATTCTACTTCGAGC
MTT5'RV	GATCTGGATCCATATGTTTAAGTTTAGTATTATTATTTATTTTATTAGAGC
MTT3'FW	CATATGGGATCCAGATCTATATGTTAATTAAAATTTAAAATATGTTGATG
MTT3'RVSpe	GCACTAGTAAATATCCAAAGATGTTTATGAG
MCSfw	GATCCTAGTAGCCTAGGTAGTAGGCTAGCTAGTAGCAATTGCCTGCAGGACGCGTGATGAT
MCSrev	GATCATCATCACGCGTCCTGCAGGCAATTGCTACTAGCTAGCCTACTACCTAGGCTACTAG
H4fw	CGCAAGCTTGAGGTCGACGGTATCGATAAGCTTGATATCTTCAAAGTATGG
H4rev	GCGTGTAAACCATCTTGTTCAATCATTTTTGTAAGTTTTTATAATCTTATTTGTTTTTCTATTTATTG
Neo4FW	AGACAATTTATTTCTAAAAAATATTTAAAAATAAAAAATAATAAGGG
Neo4RV	TGCATTTTTCCAGTAAAAATTTGAAAATTTAATGGC
HA-GA-Cre-NdeFW	GGAATTCCATATGTATCCTTATGATGTTCCTGATTATGCTGGTGCTAGCAACCTGCTGACCGTTCATC
Cre-MluRV	CGACGCGTCGTCAATCGCCATCTTCCAGCAGGC
LoxNeoFWXho	GCGCTCGAGATAACTTCGTATAATGTATGCTATACGAAGTTATGGGCTGCAGGAATTCGATAGAC
LoxNeoRV	CACCCTTAGAAACCATATAACTTCGTATAGCATACATTATACGAAGTTATGGGCTGCATTTTTCCAG
LoxGFPFW	GCTATACGAAGTTATATGGTTTCTAAGGGTGAAGAACTTTTCACTGG
LoxGFPRVBam	GCCGGATCCACTAGTCTTATATAATTCATCCATACC
TWI15LoxFW	GTCAGAAGATCCTTTCTATGTGTCC
TWI15LoxRVATGplus	CCTGCAGCCCATAACTTCGTATAGCATACATTATACGAAGTTATGACATGGATGTTAATTATATCGC
TWI1NGFPFW	GGATGAATTATATAAGACTAGTATGTCTAACAAAGGCCTTGTC
TWI1NGFPRV	GAGGCTAGTTTGGGTCGATGTTACC
EGFP-NtermRV	CCATAAGTAAGAGTAGTAACTAAAGTAGGCCAAGG
XbaBTU15FW	GGCGTCTAGAGTTGTTTGGATAATTAGATCTCTCTC
BTU1_5RV_SSS	ACTAGTCCCGGGGTCGACATCACCCAAATAAATACACGC
BTU1_3FW_SSS	GTCGACCCCGGGACTAGTTGAGCGAACTGAATCGGTCAGC
BTU13RVXho	GCGGCTCGAGAAGATGTGGCTATTGATGGGC
neo5_FW_Sal	CCGCGTCGACTTGATATCTTCAAAGTATGG
MTT1_MCS_RV	CGCCACTAGTAGATCTGGATCATCATCACGCGTC
pBNMB_addXhoAS	CCAAACAACTCTAGACTCGAGCGGCCGCCACCGC
pBNMB_addXhoS	GCGGTGGCGGCCGCTCGAGTCTAGAGTTGTTTGG

### Plasmid constructions

To construct pMNMM3 (Fig. 1A), which contains an expression cassette that allows inducible gene expression under the control of the *MTT1 *promoter, first, a ~2 kb region upstream of the *MTT1 *translational start codon (*MTT1*-5') and a ~1 kb region downstream of the *MTT1 *translational stop codon (*MTT1*-3') were amplified from genomic DNA of CU427 by the PCR Extender System (5-PRIME) with the combinations of primers MTT5'FWXho + MTT5'RV and MTT3'FW + MTT3'RVSpe, respectively. Then, *MTT1*-5' and *MTT1*-3' were connected by overlapping PCR with primers MTT5'FWXho and MTT3'RVSpe. The overlapping PCR produced NdeI, BamHI and BglII sites between *MTT1*-5' and *MTT1*-3'. The PCR product was cloned into the XhoI and SpeI sites of pBlueScript SK(+) vector (Stratagene) to produce pMMM. Then, the plasmid was digested with AccI, which cuts approximately in the middle of *MTT1*-5' and was blunt-ended by T4 DNA polymerase. A *neo2 *cassette (a hybrid *H4.1/neo/BTU2 *gene) was digested out from pNeo2 (Gaertig et al. 1994) by BamHI and XhoI, blunt-ended, and ligated with the AccI digested/blunt-ended pMMM, resulting in pMNMM. The insertion of *neo2 *splits *MTT1*-5' into two ~1 kb segments, named *MTT1*-5'-1 and *MTT1*-5'-2. *MTT1*-5'-2 contains the ~0.9 kb *MTT1 *promoter [12], which is sufficient to drive the gene expression in a heavy metal ion-dependent manner. Next, a multi-cloning site, including AvrII, NheI, MfeI, PstI, SbfI and MluI, was produced by inserting the annealed MCSfw and MCSrev oligo DNAs into the BamHI site of pMNMM. The resulting plasmid was named pMNMM2. We could obtain only a few paromomycin-resistant transformants using this construct and experienced difficulties with phenotypic assortments. As the *neo2 *coding sequence is derived from a bacteriophage and therefore not codon-optimized for Tetrahymena, the expression level of the Neo protein may not be sufficient to produce enough paromomycin-resistant transformants that can be assorted appropriately. Therefore, we replaced *neo2 *with *neo5*, in which the *neo *coding sequence was optimized for *Tetrahymena *codon usage [13]. To create *neo5*, a *neo4 *cassette was amplified from pNeo4 [13] by PrimeStar HS DNA Polymerase (Takara) with Neo4FW and Neo4RV and its *MTT1 *promoter was replaced using overlapping PCR with the histone H4.1 promoter, which was amplified from pMNMM1 with primers H4fw and H4rev. Then, *neo2 *from pMNMM2 was removed by SalI and SmaI and replaced with the amplified *neo5 *cassette, resulting in pMNMM3 (Fig. 1A). The DNA sequence of pMNMM3 can be found in the Additional file 1.

A Cre-recombinase (DDBJ/EMBL/GenBank AAG34515) encoding DNA, which was optimized for *Tetrahymena *codon-usage, was synthesized (MR. GENE GmbH, Regensburg, Germany) and named *cre1*. An HA sequence including a short two-amino acid linker (GA) was added at the N-terminus of *cre1 *by PCR amplifying the *cre1 *coding sequence using PrimeStar HS DNA Polymerase (Takara) with the primers HA-GA-Cre-NdeFW and Cre-MluRV. Then, this PCR product was cloned into NdeI and MluI sites of pMNMM3 to produce pMNMM3-*HA-cre1 *(Fig. 1B). The *MTT1*-5'-1-*neo5*-*MTT1*-5'-2-*HA-cre1*-*MTT1*-3' construct was excised from the vector backbone by digesting pMNMM3-*HA-cre1 *with XhoI and SpeI. The DNA sequence of pMNMM3-*HA-cre1 *can be found in the Additional file 1.

### Construction of the loxP-*neo4*-loxP-*EGFP-TWI1 *construct by PCR

First, the loxP-*neo4*-loxP sequence was generated by PCR amplifying the *neo4 *cassette with the primers LoxNeoFWXho and LoxNeoRV. These primers had loxP sequences at their 5'-termini. PrimeStar HS DNA Polymerase (Takara) was used for all PCR reactions in this section. In parallel, *EGFP* was amplified by PCR with the primers LoxGFPFW and LoxGFPRVBam using pOptiGFP as a template. pOptiGFP has a *EGFP *sequence optimized for *Tetrahymena *codon-usage (Kataoka et al. submitted with this manuscript). A short complementary sequence was designed at the 3'-terminus of loxP-*neo4*-loxP and the 5'-terminus of *EGFP*. Then, loxP-*neo4*-loxP and *EGFP *PCR products were concatenated by overlapping PCR with LoxNeoFWXho and LoxGFPRVBam. The resulting loxP-*neo4*-loxP-*EGFP *was cloned into the BamHI and XhoI sites of pBlueScript SK(+) to create ploxP-*neo4*-loxP-*EGFP*.

The loxP-*neo4*-loxP-*EGFP-TWI1 *construct (see Fig. 3A) was generated by PCR. The 5'-flanking and N-terminal regions of the *TWI1 *gene were amplified using the primers *TWI1*5LoxFW + *TWI1*5LoxRVATGplus and *TWI1 *NGFPFW + TWI1NGFPRV, respectively, resulting in *TWI1*-5F and *TWI1*-N. Also, loxP-*neo4*-loxP-*EGFP *was excised from ploxP-*neo4*-loxP-*EGFP *using BamHI and XhoI. This fragment had overlapping sequences with the 3' terminus of *TWI1*-5F and with the 5'- terminus of *TWI1*-N, respectively. Finally, the three DNA segments, *TWI1*-5F, loxP-*neo4*-loxP-*EGFP *and *TWI1*-N were combined by overlapping PCR using *TWI1*5LoxFW and *TWI1 *NGFPRV. The PCR product loxP-*neo4*-loxP-*EGFP-TWI1 *was purified and used directly for the transformation of *Tetrahymena*.

### Construction of *Tetrahymena *strains CRE556 and loxP-*neo4*-loxP-*EGFP-TWI1*

Biolistic gun transformation was performed as described [2] to introduce the constructs into the macronucleus by homologous recombination. The B2086 and CU428 wild-type strains were transformed with the digested pMNMM3-*HA-cre1 *and the loxP-*neo4*-loxP-*EGFP-TWI1 *PCR products, respectively. The transformants were selected using 100 μg/mL paromomycin. To select loxP-*neo4*-loxP-*EGFP-TWI1 *possessing cells, 1 μg/mL cadmium chloride was added to the medium because *neo *expression is controlled by the cadmium-dependent *MTT1 *promoter in *neo4*. In contrast, cells transformed with the MNMM3-*HA-cre1 *construct were selected without cadmium due to the two following reasons: 1) the expression of *neo *in the *neo5 *cassette is driven by the constitutive histone H4.1 promoter and thus is not dependent on cadmium ions, and 2) the presence of cadmium ions induces the expression of *HA-cre1 *from the *MTT1 *promoter in this construct and causes the suppression of cell growth (see Fig. 2C). The endogenous *MTT1 *or *TWI1 *loci were replaced with the constructs by phenotypic assortment and selection using increasing concentrations of paromomycin. One of the established strains, CRE556 (mating type II), was used for further studies.

### Western blotting

Whole-cell protein extracts were separated by SDS-PAGE and transferred to PVDF membranes. Blots were incubated in blocking solution (1% BSA, 1% skim milk, 0.1% Tween 20 in PBS) with 1:2,000 diluted mouse anti-HA antibody (16B12, Covance) or with 1:10,000 diluted mouse anti-β-tubulin antibody (12G10, Developmental Studies Hybridoma Bank, University of Iowa) and were visualized by incubation with a 1:10,000 dilution of HRP-conjugated anti-mouse IgG antibody (Jackson ImmunoResearch) in the blocking solution followed by a chemiluminescent reaction (GE Healthcare).

### Immunofluorescence staining

Cells were fixed in 3.7% formaldehyde and 0.5% Triton-X 100 for 30 min at RT, resuspended in 3.7% formaldehyde and 3.4% sucrose, and dried on poly-L-lysine (Sigma)-coated cover slips. The samples were blocked for 1 hr at 37°C with 3% BSA (Sigma), 10% normal goat serum (Invitrogen), and 0.1% Tween 20 in PBS followed by incubation in blocking solution containing a 1:2,000 dilution of mouse anti-HA antibody (16B12, Covance) for 2 hr at RT. After washes with PBS containing 0.1% Tween 20, samples were incubated with a 1:2,000 dilution of anti-mouse antibody conjugated with Alexa 488 (Invitrogen) for 1 hr at RT. The samples were washed, incubated with 10 ng/mL DAPI (Sigma) in PBS, mounted with ProLong Gold (Invitrogen), and observed by fluorescence microscopy.

### *Tetrahymena *cell growth assay

Late log cultures of B2086 and CRE556 were diluted to 5 × 10^3 ^cells/mL in a fresh 1× SPP medium with or without 1 μg/mL CdCl_2 _and cultured at 30°C with rotation at 100 rpm. Every 5 hours, cells were counted to monitor cell growth using a model ZB1 Coulter counter (Coulter Electronics Inc).

### Construction of *Tetrahymena *strains expressing *HA-cre1 *from *BTU1 *locus

To express *HA-cre1 *from the BTU1 locus, pBNMB-*HA-cre1 *was created. First, a ~0.8 kb upstream (*BTU1*_5') and a ~0.8 kb downstream (*BTU1*_3') region of the *BTU1 *gene were amplified from the genomic DNA of B2086 by the PrimeStar HS DNA Polymerase (Takara) with the combinations of primers XbaBTU15FW + BTU1_5RV_SSS and BTU1_3FW_SSS + BTU13RVXho, respectively. Then, two PCR products were connected by overlapping PCR with primers XbaBTU15FW and BTU13RVXho. The overlapping PCR produced SalI, SmaI and SpeI sites between *BTU1_*5' and *BTU1_*3'. The PCR product was cloned into the XbaI and XhoI sites of pBlueScript SK(+) vector (Stratagene) to produce pBB. The *neo5*-*MTT1*-5'-2 segment was amplified from pMNMM3 by the PrimeStar HS DNA Polymerase with primers neo5_FW_Sal and MTT1_MCS_RV and cloned into the SalI and SpeI site of pBB. Then, an XhoI site was introduced between XbaI and NotI sites by site-directed mutagenesis with primers pBNMB_addXhoS and pBNMB_addXhoAS to produce pBNMB. *neo5*-*MTT1*-5'-2-*HA-cre *segment of pMNMM3-*HA-cre1 *was excised out by SalI and MluI and cloned into the SalI and MluI site of pBNMB to produce pBNMB-*HA-cre1*. The plasmid map and the DNA sequence of pBNMB-*HA-cre1 *can be found in the Additional file 1.

The CU427 wild-type strain was transformed with the *BTU1*-5'-*neo5*-*MTT1*-5'-2-*HA-cre1*-*BTU1*-3' construct which was digested out from pBNMB-*HA-cre1 *and the transformants were selected using 100 μg/mL paromomycin. The endogenous *BTU1 *loci were replaced with the construct by phenotypic assortment and selection using increasing concentrations of paromomycin. Six strains were selected for further studies.

### Induction of Cre-mediated loxP recombination

For the experiment shown in Fig. 2A, exponentially growing B2086 or CRE556 cells were cultured in 1× SPP medium with or without 1 μg/mL CdCl_2 _for 1.5 hr, or starved B2086 or CRE556 cells were cultured in 10 mM Tris (pH 7.5) with or without 50 ng/mL CdCl_2 _for 1.5 hr.

For the experiment shown in Fig. 2B, CRE556 and loxP-*neo4*-loxP-*EGFP-TWI1 *strains, both pre-starved over night in 10 mM Tris (pH 7.5) were mated and 50 ng/mL CdCl_2 _was added to the culture at 3.5 hr post-mixing (hpm). At the times indicated, cells were collected for immunofluorescence staining.

For the experiment shown in Fig. 3B, starved CRE556 cells were pre-treated with 50 ng/mL CdCl_2 _for 1.5 hours and then mated with starved loxP-*neo4*-loxP-*EGFP-TWI1 *cells in 10 mM Tris (pH 7.5) in the presence of 25 ng/mL CdCl_2_. At 2, 4, 6 and 8 hpm, genomic DNA was extracted for loxP excision analysis.

For the experiment shown in Fig. 4B, starved CRE556 cells were pre-treated with 50 ng/mL CdCl_2 _in 10 mM Tris (pH 7.5) for 1.5 hr and then mated with pre-starved loxP-*neo4*-loxP-*EGFP-TWI1 *strains in the presence of 25 ng/mL CdCl_2_. At 2 hpm, single mating pairs were isolated into drops of 1× SPP medium. Cells were observed about every 2 hr until 6 hpm; then, cells were cloned into fresh drops of 1× SPP medium, in cases where pairs had separated. Cells were cultured for 2 days at 30°C and established clones were cultured in 1 mL 1× SPP medium for ~24 hr. The cells were then inoculated into 1× SPP medium containing 1 μg/mL CdCl_2_. Clones growing at normal speed in this medium were chosen as candidates for loxP-*neo4*-loxP-*EGFP-TWI1 *strain derived cells. This selection is based on the fact that HA-Cre1p expression severely suppresses cell growth (see text for details). The selected strains were used for loxP excision analysis. These procedures are schematically drawn in Fig. 4A.

### loxP excision analysis by PCR

Cells were lysed in guanidine solution (4 M guanidine thiocyanate, 0.5% N-lauroyl sarcosine sodium, 25 mM Tris-HCl pH 8.0, 0.1 M 2-mercaptoethanol) and genomic DNA was extracted by conventional extraction with phenol/chloroform (1:1) and precipitated with isopropanol. The loxP-*neo4*-loxP-*EGFP-TWI1 *locus or the *neo4*-excised loxP-*EGFP-TWI1 *locus was detected using the PCR Extender System (5-PRIME) with the primers TWI15LoxFW and EGFP-NtermRV.

### Observation of EGFP-Twi1p

loxP-*EGFP-TWI1 *cells were mated with the wild-type B2086 strain. Cells were fixed and stored in 25% methanol and 10% formaldehyde over night at 4°C. The samples were incubated with 10 ng/mL DAPI and observed by fluorescence microscopy.

## Authors' contributions

CJB and KM designed the project; CJB, AV and KM performed experiments; CJB and KM analyzed the data and wrote the paper. All authors read and approved the final manuscript.

## Supplementary Material

Additional file 1 **Supplementary Figure S1 and plasmid DNA sequences**. Supplementary Figure S1 describing construction and analyses of a *Tetrahymena *strain expressing Cre-recombinase from *BTU1 *locus, and DNA sequences of pMNMM3, pMNMM3-HA-cre1 and pBNMB-HA-cre1 Click here for file
