# Supplementary material for: Establishment of a Cre/loxP recombination system for N-terminal epitope tagging of genes in Tetrahymena
Source: BMC Microbiol. 2010 Jul 13;10:191. doi: 10.1186/1471-2180-10-191 (PMC2912859; doi:10.1186/1471-2180-10-191)
Supplement: Additional file 1 — Supplementary Figure S1 and plasmid DNA sequences. Supplementary Figure S1 describing construction and analyses of a Tetrahymena strain expressing Cre-recombinase from BTU1 locus, and DNA sequences of pMNMM3, pMNMM3-HA-cre1 and pBNMB-HA-cre1 [file 1471-2180-10-191-S1.PDF]

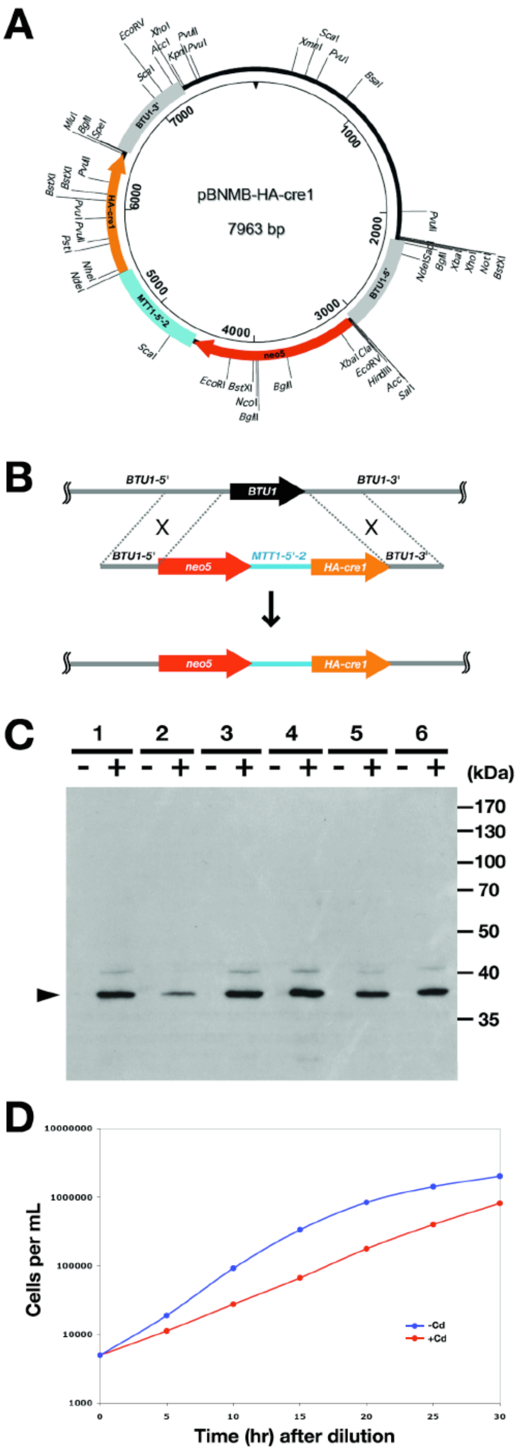

Figure S1. Construction and analyses of a *Tetrahymena* strain expressing Cre-recombinase from *BTU1* locus

(A) Plasmid map of pBNMB-*HA-cre1*. (B) Diagrams of the wild-type *BTU1,* BNMB-*HA-cre1* construct and BNMB-*HA-cre1* locus. (C) Expression of HA-Cre1p in 6 independent BNMB-HA-cre1 strains is induced by the presence of cadmium ions. BNMB-*HA-cre1* strains were cultured in the nutrient-rich 1x SPP medium and were treated with (+) or without (-) 1 µg/mL $CdCl_2$. HA-Cre1p was detected by western blotting using an anti-HA antibody. The position of HA-Cre1p (39.7 kDa) is marked by an arrowhead. (D) Expression of HA-Cre1p from the *BTU1* locus suppresses growth of *Tetrahymena.* BNMB-HA-cre1#4 cells were diluted to 5,000 cells/mL with 1x SPP medium with or without 1 µg/mL $CdCl_2$. At indicated time after dilution, cells were counted to monitor cell growth.

DNA sequence of pMNMM3

CTAAATTGTAAGCGTTAATATTTTGTTAAAATTCGCGTTAAATTTTTGTTAAATCAGCTCATTTTTTAA
CCAATAGGCCGAAATCGGCAAAATCCCTTATAAATCAAAAGAATAGACCGAGATAGGGTTGAGTGTTGT
TCCAGTTTGGAACAAGAGTCCACTATTAAAGAACGTGGACTCCAACGTCAAAGGGCGAAAAACCGTCTA
TCAGGGCGATGGCCCACTACGTGAACCATCACCCTAATCAAGTTTTTTGGGGTCGAGGTGCCGTAAAGC
ACTAAATCGGAACCCTAAAGGGAGCCCCCGATTTAGAGCTTGACGGGGAAAGCCGGCGAACGTGGCGAG
AAAGGAAGGGAAGAAAGCGAAAGGAGCGGGCGCTAGGGCGCTGGCAAGTGTAGCGGTCACGCTGCGCGT
AACCACCACACCCGCCGCGCTTAATGCGCCGCTACAGGGCGCGTCCCATTCGCCATTCAGGCTGCGCAA
CTGTTGGGAAGGGCGATCGGTGCGGGCCTCTTCGCTATTACGCCAGCTGGCGAAAGGGGGATGTGCTGC
AAGGCGATTAAGTTGGGTAACGCCAGGGTTTTCCCAGTCACGACGTTGTAAAACGACGGCCAGTGAGCG
CGCGTAATACGACTCACTATAGGGCGAATTGGGTACCGGGCCCCCCCTCGAGAGTCTTTGCATTCTACT
TCGAGCAAACTATAAAAATATTTGAGCATACTTTCTAAATCTAATTTTAAAAGATATTTCTTAAAGTCT
TTAAAGTTAGTTTTTTTTATTGAAGAATAATTAGCTTTAATCAAGTAAAATGTTTTATTGTTAATTTAT
TTAATTAGATTTATTTTATAAGTTATTTTGGCTATGGTTAGCAAATTATATCTTTAAATCCTTTTTCAT
ACTTATTTTGTATATTCAAAATCTCAACAAAAGCTAATTTCTACTTTTTAATTAATTAATTTCTACTAA
TTTAGTAAAAAATAAAAATTTGCAAATTTTTTAATTAATTAAAAATCCTTTGTAACGTTTTGATGTAAT
TTTAAGAGTCCTTGGTCTTAACACTAAAAACTTATTTAAAGAAAAATAAATCAATAATGCAAATTTATT
TTATTCAAGGCTTGCTTTTAAAGCATTAAATAGGATTAAACAATATAAATTTTAGCTATTTAAATTAAA
TTTATTATTTTTAATGATTAAAATTATTAAACTTGAATAAATGATCCTAATTCTTTTTGTTATTTATAT
TGGAATAACATTTAAGTAAATCAACATAAAAATGACTTTAATACAATTTACTATTCTTCATAGTAGTTG
CTTGAGCTTTTGAAGTATGAATTAGGTTTAAATATCATATTATTTTAGCTGTTTAAATTAAATTTATTA
TTTTTGATGATTAAAATAATTCAGATTTTATGAATGATACTACTTATTTTAAATTTCATGTAAAAATAA
TATTAGTGATGGTATTAAAAATATAATTAGATTTTTTAAAAATTGTTTGGAAATAAGTACTTAATTCCA
AATTTCTTTTAATTTTTAAGAATTAAATTAGTTTATTTTTATATATTAATTCACTAAATAAAAAGATAA
GTAATATATTTAGTGCACAATGTTTGAATGTTTTTTTGATAAATTTGTAAATTAAATCTATTTAAGTCA
GTAGAATTTTAGATATTTTCATAGATTTTTTAATAAGTAGTCGAGGTCGACGGTATCGATAAGCTTGAT
ATCTTCAAAGTATGGATTAATTATTTCAAATTATTAGAAGGTAATTAATCTGCATAAATTCAAAACTAT
AAAAATAAAACATTAAAATTAATTCAACCTTATTGAAGCATCAAAATCTGAATCTCTAGAAAGACTGAT
TCTGATTGGATAATTTTTCGGCGCTAAGGATTTTGGATTAAAGAAAATTAGATTTAATTATTAATCATG
ATTTGAATAGGATAGCAAGAATATTTGTTTGGTTTAAAAGGGAAAGCGGGTAATTATCAAAAATTTATA
AATAATTTTAAAACAATAAATAGAAAAACAAATAAGATTATAAAAACTTACAAAAATGATTGAACAAGA
TGGTTTACACGCTGGTTCTCCCGCCGCTTGGGTCGAAAGACTTTTCGGTTATGACTGGGCTCAACAAAC
CATCGGTTGCTCTGATGCCGCCGTCTTCCGTCTTTCTGCTCAAGGTCGTCCTGTTCTTTTCGTCAAGAC
CGACCTTTCTGGTGCCCTTAATGAACTTCAAGATGAAGCTGCCCGTCTTTCTTGGCTTGCCACCACCGG
TGTTCCTTGCGCTGCTGTCCTTGACGTTGTCACTGAAGCCGGTAGAGACTGGCTTCTTTTAGGTGAAGT
CCCCGGTCAAGATCTTCTTTCTTCTCACCTTGCTCCTGCCGAAAAAGTTTCTATCATGGCTGATGCTAT
GCGTCGTCTTCATACCCTTGATCCCGCTACCTGCCCTTTCGACCACCAAGCCAAACATCGTATCGAACG
TGCTCGTACTCGTATGGAAGCCGGTCTTGTCGATCAAGATGATCTTGACGAAGAACATCAAGGTCTTGC
CCCTGCCGAACTTTTCGCCAGACTTAAGGCCCGTATGCCCGACGGTGAAGATCTTGTCGTCACCCATGG
TGATGCCTGCTTACCCAATATCATGGTTGAAAATGGTCGTTTTTCTGGTTTCATCGACTGTGGTCGTCT
TGGTGTCGCCGACCGTTATCAAGATATTGCCTTAGCTACCCGTGATATTGCTGAAGAACTTGGTGGTGA
ATGGGCTGACCGTTTCCTTGTCCTTTACGGTATCGCCGCTCCCGATTCTCAACGTATCGCCTTCTATCG
TCTTCTTGACGAATTCTTCTGAGATCCTTAAATTAAAAATTCAATATATATTTACAAACTTTCATATAA
AATAAATATATTATATAAAATTAATTTTTAGTGTATTATATTAACATTAAAGCACCAAAAAAACGTGTT
AATATACTACTATAAAATATAATTTATTCCAAATTGACTAAAATCATTATTTTACAACTCATTTGTATA
TATATTTTATGTCAATTATTTTTTTTAACTTTCTAAAAAAAAAAATTCCTCTTCACATACATGTTAGCT
CTTAAAAATTTGTCTGCAAATCCAATAATAATATTTTTTTTTGCCATTAAATTTTCAAATTTTTACTGG
AAAAATGCAGCCCGGGGGATCAGACAATTTATTTCTAAAAAATATTTAAAAATAAAAAATAATAAGGGT
TTTGAATAACTCCTTTAATTTAAATACACATTTTTAAATTTTTTTTAGCTCTTTAAATATTCATAAAAA
TAAAAAATAACTAACTAAAAATAAATAAAAAGATAATAATGATTAAAGGTATAATACTGAATAAGAAAA
AACATAATAGAGTACTTATTTTTTATATCACTATTTTTAATATCTTGAAAGCAAAACTTTTTTATATAT
CTTAAAATATATTGTATCGTTTATTCAATTATTTTCTTTAAATTTCAAATATATTGATAAAAAAGATGA
CATGTTTTTTAAAGAAAACATGAAATATAAAATAGATAAATATCAATTATTTTATTTATTAAATATATA
AGCTGCTCAAAACATAGCTCATTCATCAATTATAATATGTGAATCATTAATTTTCAAAATATTACTCAT
TATTTAGGCTATCATTTATTTTTTATTTTCAATTATCCGTTTCTATTATATTTTAATATTAAGTTGTGA
TTCTTGAATTTTGTGTCATGAATTATTTGTAAATCTTTTTATTTCTGATAAAAAATATAAATTGATTGA
CTCATGATTTAAATCATGAGTCAACCTAACTAATTTTCAAAATTCTTCTATTCTAAAATATAGATGTGA
TTCTTGAATCTCTCTTGAATATAAAGTAATTTTTTATATTTCTGATATAATTCTTAGCTACGTGATTCA
CGATTTATGCAATGATCCATATAAAATAATGTAAATAGTGTATATATATATATTCGTCTTTTTTATTCT
TTATATAATTTAAAAAAATTAAAAAAATTTAATAAAGCTCTAATAAAATAAATAATAATACTAAACTTA

AACATATGGGATCCTAGTAGCCTAGGTAGAGGCTAGCTAGTAGCAATTGCCTGCAGGACGCGTGATGAT
GATCCAGATCTATATGTTAATTAAAATTTAAAATATGTTGATGTTGTAATTCATAATTGCATAAAATCA
GTTTCGTATTTTATTAAAAACTTTAATATTATCATCCTAAAATTTATTAAAAAAAAACTGTTATTAATAA
ATCAATAATTATCGCTATTTCATAGATTTTTTTAAATGCAAAAGCAAGTATGTTATTTAAATTAAGTAA
ACATTGATTGAAAGTATTATCTACAGAAAAAAAAAAATATATAATTTACTTTTTAATCAATTTAATTGGA
AATTATTTTATATTTTTTTAAAAATGAAAAAACATATATATTTATAATAAACATTCAAATTTATTTTACA
AAATTTATATAAAAATCTTTAATAATTACAATTAATTGAATTCAATCTGTAAATATAAAATTACACCAT
ACATTTATATTAACAATGACTACTTTTAGAAAAAAATTTAAAAAAAAATAAATTAATGTTTTATTTTAA
ATTTTGGTAAAAATACTCAACTTATTTGAATTTAGAAAATGTAAAATCAAATAAATTATTTTAATTTAA
TGAAAAAGTTTTTTTGAATTAGTAAATATTATCAACTAAAAAACAAACTGAAAAACCATATTTGGTAAT
TATTACTTTTGCATAAAGAAATTTAATAATGTAAATGAAATGAATATCTCTAAAAATTATATAAAAAAT
ATCAATTTTAAGCTAATTGTCAATTAATAAATGCTAAAATGAAAATAAATCTAAATAAAAAAATATCTA
TGTTCATATTTGCATAAAATAAATTGTTTGTTGCAGTTTTTAGTTGCAATTAAAACTTTAAATATAAAA
ATATATTTATTATAATTTAATTTTAAGACCTACTTATTTTTTTTAAATTATATTTATTAAAAGAATTTA
ATTTATTTGTGAATTAATTTTAATATGTATTTATCTCATAAACATCTTTGGATATTTACTAGTTCTAGA
GCGGCCGCCACCGCGGTGGAGCTCCAGCTTTTGTTCCCTTTAGTGAGGGTTAATTGCGCGCTTGGCGTA
ATCATGGTCATAGCTGTTTCCTGTGTGAAATTGTTATCCGCTCACAATTCCACACAACATACGAGCCGG
AAGCATAAAGTGTAAAGCCTGGGGTGCCTAATGAGTGAGCTAACTCACATTAATTGCGTTGCGCTCACT
GCCCGCTTTCCAGTCGGGAAACCTGTCGTGCCAGCTGCATTAATGAATCGGCCAACGCGCGGGGAGAGG
CGGTTTGCGTATTGGGCGCTCTTCCGCTTCCTCGCTCACTGACTCGCTGCGCTCGGTCGTTCGGCTGCG
GCGAGCGGTATCAGCTCACTCAAAGGCGGTAATACGGTTATCCACAGAATCAGGGGATAACGCAGGAAA
GAACATGTGAGCAAAAGGCCAGCAAAAGGCCAGGAACCGTAAAAAGGCCGCGTTGCTGGCGTTTTTCCA
TAGGCTCCGCCCCCCTGACGAGCATCACAAAAATCGACGCTCAAGTCAGAGGTGGCGAAACCCGACAGG
ACTATAAAGATACCAGGCGTTTCCCCCTGGAAGCTCCCTCGTGCGCTCTCCTGTTCCGACCCTGCCGCT
TACCGGATACCTGTCCGCCTTTCTCCCTTCGGGAAGCGTGGCGCTTTCTCATAGCTCACGCTGTAGGTA
TCTCAGTTCGGTGTAGGTCGTTCGCTCCAAGCTGGGCTGTGTGCACGAACCCCCCGTTCAGCCCGACCG
CTGCGCCTTATCCGGTAACTATCGTCTTGAGTCCAACCCGGTAAGACACGACTTATCGCCACTGGCAGC
AGCCACTGGTAACAGGATTAGCAGAGCGAGGTATGTAGGCGGTGCTACAGAGTTCTTGAAGTGGTGGCC
TAACTACGGCTACACTAGAAGGACAGTATTTGGTATCTGCGCTCTGCTGAAGCCAGTTACCTTCGGAAA
AAGAGTTGGTAGCTCTTGATCCGGCAAACAAACCACCGCTGGTAGCGGTGGTTTTTTTGTTTGCAAGCA
GCAGATTACGCGCAGAAAAAAAGGATCTCAAGAAGATCCTTTGATCTTTTCTACGGGGTCTGACGCTCA
GTGGAACGAAAACTCACGTTAAGGGATTTTGGTCATGAGATTATCAAAAAGGATCTTCACCTAGATCCT
TTTAAATTAAAAATGAAGTTTTAAATCAATCTAAAGTATATATGAGTAAACTTGGTCTGACAGTTACCA
ATGCTTAATCAGTGAGGCACCTATCTCAGCGATCTGTCTATTTCGTTCATCCATAGTTGCCTGACTCCC
CGTCGTGTAGATAACTACGATACGGGAGGGCTTACCATCTGGCCCCAGTGCTGCAATGATACCGCGAGA
CCCACGCTCACCGGCTCCAGATTTATCAGCAATAAACCAGCCAGCCGGAAGGGCCGAGCGCAGAAGTGG
TCCTGCAACTTTATCCGCCTCCATCCAGTCTATTAATTGTTGCCGGGAAGCTAGAGTAAGTAGTTCGCC
AGTTAATAGTTTGCGCAACGTTGTTGCCATTGCTACAGGCATCGTGGTGTCACGCTCGTCGTTTGGTAT
GGCTTCATTCAGCTCCGGTTCCCAACGATCAAGGCGAGTTACATGATCCCCCATGTTGTGCAAAAAAGC
GGTTAGCTCCTTCGGTCCTCCGATCGTTGTCAGAAGTAAGTTGGCCGCAGTGTTATCACTCATGGTTAT
GGCAGCACTGCATAATTCTCTTACTGTCATGCCATCCGTAAGATGCTTTTCTGTGACTGGTGAGTACTC
AACCAAGTCATTCTGAGAATAGTGTATGCGGCGACCGAGTTGCTCTTGCCCGGCGTCAATACGGGATAA
TACCGCGCCACATAGCAGAACTTTAAAAGTGCTCATCATTGGAAAACGTTCTTCGGGGCGAAAACTCTC
AAGGATCTTACCGCTGTTGAGATCCAGTTCGATGTAACCCACTCGTGCACCCAACTGATCTTCAGCATC
TTTTACTTTCACCAGCGTTTCTGGGTGAGCAAAAACAGGAAGGCAAAATGCCGCAAAAAAGGGAATAAG
GGCGACACGGAAATGTTGAATACTCATACTCTTCCTTTTTCAATATTATTGAAGCATTTATCAGGGTTA
TTGTCTCATGAGCGGATACATATTTGAATGTATTTAGAAAAATAAACAAATAGGGGTTCCGCGCACATT
TCCCCGAAAAGTGCCAC

DNA sequence of pMNMM3-HA-cre1

CTAAATTGTAAGCGTTAATATTTTGTTAAAATTCGCGTTAAATTTTTGTTAAATCAGCTCATTTTTTAA
CCAATAGGCCGAAATCGGCAAAATCCCTTATAAATCAAAAGAATAGACCGAGATAGGGTTGAGTGTTGT
TCCAGTTTGGAACAAGAGTCCACTATTAAAGAACGTGGACTCCAACGTCAAAGGGCGAAAAACCGTCTA
TCAGGGCGATGGCCCACTACGTGAACCATCACCCTAATCAAGTTTTTTGGGGTCGAGGTGCCGTAAAGC
ACTAAATCGGAACCCTAAAGGGAGCCCCCGATTTAGAGCTTGACGGGGAAAGCCGGCGAACGTGGCGAG
AAAGGAAGGGAAGAAAGCGAAAGGAGCGGGCGCTAGGGCGCTGGCAAGTGTAGCGGTCACGCTGCGCGT
AACCACCACACCCGCCGCGCTTAATGCGCCGCTACAGGGCGCGTCCCATTCGCCATTCAGGCTGCGCAA
CTGTTGGGAAGGGCGATCGGTGCGGGCCTCTTCGCTATTACGCCAGCTGGCGAAAGGGGGATGTGCTGC
AAGGCGATTAAGTTGGGTAACGCCAGGGTTTTCCCAGTCACGACGTTGTAAAACGACGGCCAGTGAGCG
CGCGTAATACGACTCACTATAGGGCGAATTGGGTACCGGGCCCCCCCTCGAGAGTCTTTGCATTCTACT
TCGAGCAAACTATAAAAATATTTGAGCATACTTTCTAAATCTAATTTTAAAAGATATTTCTTAAAGTCT

TTAAAGTTAGTTTTTTTTATTGAAGAATAATTAGCTTTAATCAAGTAAAATGTTTTATTGTTAATTTAT
TTAATTAGATTTATTTTATAAGTTATTTTGGCTATGGTTAGCAAATTATATCTTTAAATCCTTTTTCAT
ACTTATTTTGTATATTCAAAATCTCAACAAAAGCTAATTTCTACTTTTTAATTAATTAATTTCTACTAA
TTTAGTAAAAAATAAAAATTTGCAAATTTTTTAATTAATTAAAAATCCTTTGTAACGTTTTGATGTAAT
TTTAAGAGTCCTTGGTCTTAACACTAAAAACTTATTTAAAGAAAAATAAATCAATAATGCAAATTTATT
TTATTCAAGGCTTGCTTTTAAAGCATTAAATAGGATTAAACAATATAAATTTTAGCTATTTAAATTAAA
TTTATTATTTTTAATGATTAAAATTATTAAACTTGAATAAATGATCCTAATTCTTTTTGTTATTTATAT
TGGAATAACATTTAAGTAAATCAACATAAAAATGACTTTAATACAATTTACTATTCTTCATAGTAGTTG
CTTGAGCTTTTGAAGTATGAATTAGGTTTAAATATCATATTATTTTAGCTGTTTAAATTAAATTTATTA
TTTTTGATGATTAAAATAATTCAGATTTTATGAATGATACTACTTATTTTAAATTTCATGTAAAAATAA
TATTAGTGATGGTATTAAAAATATAATTAGATTTTTTAAAAATTGTTTGGAAATAAGTACTTAATTCCA
AATTTCTTTTAATTTTTAAGAATTAAATTAGTTTATTTTTATATATTAATTCACTAAATAAAAAGATAA
GTAATATATTTAGTGCACAATGTTTGAATGTTTTTTTGATAAATTTGTAAATTAAATCTATTTAAGTCA
GTAGAATTTTAGATATTTTCATAGATTTTTTAATAAGTAGTCGAGGTCGACGGTATCGATAAGCTTGAT
ATCTTCAAAGTATGGATTAATTATTTCAAATTATTAGAAGGTAATTAATCTGCATAAATTCAAAACTAT
AAAAATAAAACATTAAAATTAATTCAACCTTATTGAAGCATCAAAATCTGAATCTCTAGAAAGACTGAT
TCTGATTGGATAATTTTTCGGCGCTAAGGATTTTGGATTAAAGAAAATTAGATTTAATTATTAATCATG
ATTTGAATAGGATAGCAAGAATATTTGTTTGGTTTAAAAGGGAAAGCGGGTAATTATCAAAAATTTATA
AATAATTTTAAAACAATAAATAGAAAAACAAATAAGATTATAAAAACTTACAAAAATGATTGAACAAGA
TGGTTTACACGCTGGTTCTCCCGCCGCTTGGGTCGAAAGACTTTTCGGTTATGACTGGGCTCAACAAAC
CATCGGTTGCTCTGATGCCGCCGTCTTCCGTCTTTCTGCTCAAGGTCGTCCTGTTCTTTTCGTCAAGAC
CGACCTTTCTGGTGCCCTTAATGAACTTCAAGATGAAGCTGCCCGTCTTTCTTGGCTTGCCACCACCGG
TGTTCCTTGCGCTGCTGTCCTTGACGTTGTCACTGAAGCCGGTAGAGACTGGCTTCTTTTAGGTGAAGT
CCCCGGTCAAGATCTTCTTTCTTCTCACCTTGCTCCTGCCGAAAAAGTTTCTATCATGGCTGATGCTAT
GCGTCGTCTTCATACCCTTGATCCCGCTACCTGCCCTTTCGACCACCAAGCCAAACATCGTATCGAACG
TGCTCGTACTCGTATGGAAGCCGGTCTTGTCGATCAAGATGATCTTGACGAAGAACATCAAGGTCTTGC
CCCTGCCGAACTTTTCGCCAGACTTAAGGCCCGTATGCCCGACGGTGAAGATCTTGTCGTCACCCATGG
TGATGCCTGCTTACCCAATATCATGGTTGAAAATGGTCGTTTTTCTGGTTTCATCGACTGTGGTCGTCT
TGGTGTCGCCGACCGTTATCAAGATATTGCCTTAGCTACCCGTGATATTGCTGAAGAACTTGGTGGTGA
ATGGGCTGACCGTTTCCTTGTCCTTTACGGTATCGCCGCTCCCGATTCTCAACGTATCGCCTTCTATCG
TCTTCTTGACGAATTCTTCTGAGATCCTTAAATTAAAAATTCAATATATATTTACAAACTTTCATATAA
AATAAATATATTATATAAAATTAATTTTTAGTGTATTATATTAACATTAAAGCACCAAAAAAACGTGTT
AATATACTACTATAAAATATAATTTATTCCAAATTGACTAAAATCATTATTTTACAACTCATTTGTATA
TATATTTTATGTCAATTATTTTTTTTAACTTTCTAAAAAAAAAAATTCCTCTTCACATACATGTTAGCT
CTTAAAAATTTGTCTGCAAATCCAATAATAATATTTTTTTTTGCCATTAAATTTTCAAATTTTTACTGG
AAAAATGCAGCCCGGGGGATCAGACAATTTATTTCTAAAAAATATTTAAAAATAAAAAATAATAAGGGT
TTTGAATAACTCCTTTAATTTAAATACACATTTTTAAATTTTTTTTAGCTCTTTAAATATTCATAAAAA
TAAAAAATAACTAACTAAAAATAAATAAAAAGATAATAATGATTAAAGGTATAATACTGAATAAGAAAA
AACATAATAGAGTACTTATTTTTTATATCACTATTTTTAATATCTTGAAAGCAAAACTTTTTTATATAT
CTTAAAATATATTGTATCGTTTATTCAATTATTTTCTTTAAATTTCAAATATATTGATAAAAAAGATGA
CATGTTTTTTAAAGAAAACATGAAATATAAAATAGATAAATATCAATTATTTTATTTATTAAATATATA
AGCTGCTCAAAACATAGCTCATTCATCAATTATAATATGTGAATCATTAATTTTCAAAATATTACTCAT
TATTTAGGCTATCATTTATTTTTTATTTTCAATTATCCGTTTCTATTATATTTTAATATTAAGTTGTGA
TTCTTGAATTTTGTGTCATGAATTATTTGTAAATCTTTTTATTTCTGATAAAAAATATAAATTGATTGA
CTCATGATTTAAATCATGAGTCAACCTAACTAATTTTCAAAATTCTTCTATTCTAAAATATAGATGTGA
TTCTTGAATCTCTCTTGAATATAAAGTAATTTTTTATATTTCTGATATAATTCTTAGCTACGTGATTCA
CGATTTATGCAATGATCCATATAAAATAATGTAAATAGTGTATATATATATATTCGTCTTTTTTATTCT
TTATATAATTTAAAAAAATTAAAAAAATTTAATAAAGCTCTAATAAAATAAATAATAATACTAAACTTA
AACATATGTATCCTTATGATGTTCCTGATTATGCTGGTGCTAGCAACCTGCTGACCGTTCATCAGAATC
TGCCGGCGCTGCCGGTGGATGCCACCAGCGATGAAGTGCGCAAAAACCTGATGGATATGTTTCGTGATC
GTCAGGCCTTCAGCGAACATACCTGGAAAATGCTGCTGAGCGTGTGCCGTAGCTGGGCCGCGTGGTGCA
AACTGAACAACCGTAAATGGTTTCCGGCGGAACCGGAAGATGTGCGTGATTATCTGCTGTATCTGCAGG
CGCGTGGCCTGGCCGTGAAAACCATCCAGCAGCACCTGGGTCAGCTGAACATGCTGCATCGCCGTAGCG
GCCTGCCGCGCCCGAGCGATAGCAATGCGGTGAGCCTGGTGATGCGTCGTATTCGTAAAGAAAACGTGG
ATGCGGGTGAACGTGCGAAACAGGCCCTGGCGTTCGAACGCACCGATTTTGATCAGGTTCGTAGCCTGA
TGGAAAACAGCGATCGCTGCCAGGATATTCGCAACCTGGCGTTTCTGGGTATTGCGTATAATACCCTGC
TGCGCATCGCCGAAATTGCGCGCATTCGTGTTAAAGATATTAGCCGCACCGATGGCGGTCGTATGCTGA
TCCACATCGGTCGCACCAAAACCCTGGTGAGCACCGCGGGCGTGGAAAAAGCCCTGAGCCTGGGTGTGA
CCAAACTGGTGGAACGTTGGATTAGCGTGAGCGGTGTTGCGGATGATCCGAACAACTATCTGTTTTGCC
GTGTGCGCAAAAATGGCGTGGCGGCGCCGAGCGCGACCAGCCAGCTGAGCACCCGTGCCCTGGAAGGCA
TTTTTGAAGCCACCCATCGCCTGATTTATGGTGCGAAAGATGATAGCGGTCAGCGTTATCTGGCCTGGA
GCGGTCATAGCGCCCGCGTGGGTGCGGCGCGCGATATGGCGCGTGCGGGTGTGAGCATCCCGGAAATTA
TGCAGGCCGGCGGTTGGACCAATGTGAACATTGTGATGAACTATATTCGCACCCTGGATAGCGAAACCG

GTGCGATGGTGCGCCTGCTGGAAGATGGCGATTGACGACGCGTGATGATGATCCAGATCTATATGTTAA
TTAAAATTTAAAATATGTTGATGTTGTAATTCATAATTGCATAAAATCAGTTTCGTATTTTATTAAAAA
CTTTAATATTATCATCCTAAAATTTATTAAAAAAAACTGTTATTAATAAATCAATAATTATCGCTATTT
CATAGATTTTTTTAAATGCAAAAGCAAGTATGTTATTTAAATTAAGTAAACATTGATTGAAAGTATTAT
CTACAGAAAAAAAAAAATATATAATTTACTTTTTAATCAATTTAATTGGAAATTATTTTATATTTTTTAA
AAATGAAAAAACATATATATTTATAATAAACATTCAAATTTATTTTACAAAATTTATATAAAAATCTTT
AATAATTACAATTAATTGAATTCAATCTGTAAATATAAAATTACACCATACATTTATATTAACAATGAC
TACTTTTAGAAAAAAATTTAAAAAAAAATAAATTAATGTTTTATTTTAAATTTTGGTAAAAATACTCAA
CTTATTTGAATTTAGAAAATGTAAAATCAAATAAATTATTTTAATTTAATGAAAAAGTTTTTTTGAATT
AGTAAATATTATCAACTAAAAAACAAACTGAAAAACCATATTTGGTAATTATTACTTTTGCATAAAGAA
ATTTAATAATGTAAATGAAATGAATATCTCTAAAAATTATATAAAAAATATCAATTTTAAGCTAATTGT
CAATTAATAAATGCTAAAATGAAAATAAATCTAAATAAAAAAATATCTATGTTCATATTTGCATAAAAT
AAATTGTTTGTTGCAGTTTTTAGTTGCAATTAAAACTTTAAATATAAAAATATATTTATTATAATTTAA
TTTTAAGACCTACTTATTTTTTTTAAATTATATTTATTAAAAGAATTTAATTTATTTGTGAATTAATTT
TAATATGTATTTATCTCATAAACATCTTTGGATATTTACTAGTTCTAGAGCGGCCGCCACCGCGGTGGA
GCTCCAGCTTTTGTTCCCTTTAGTGAGGGTTAATTGCGCGCTTGGCGTAATCATGGTCATAGCTGTTTC
CTGTGTGAAATTGTTATCCGCTCACAATTCCACACAACATACGAGCCGGAAGCATAAAGTGTAAAGCCT
GGGGTGCCTAATGAGTGAGCTAACTCACATTAATTGCGTTGCGCTCACTGCCCGCTTTCCAGTCGGGAA
ACCTGTCGTGCCAGCTGCATTAATGAATCGGCCAACGCGCGGGGAGAGGCGGTTTGCGTATTGGGCGCT
CTTCCGCTTCCTCGCTCACTGACTCGCTGCGCTCGGTCGTTCGGCTGCGGCGAGCGGTATCAGCTCACT
CAAAGGCGGTAATACGGTTATCCACAGAATCAGGGGATAACGCAGGAAAGAACATGTGAGCAAAAGGCC
AGCAAAAGGCCAGGAACCGTAAAAAGGCCGCGTTGCTGGCGTTTTTCCATAGGCTCCGCCCCCCTGACG
AGCATCACAAAAATCGACGCTCAAGTCAGAGGTGGCGAAACCCGACAGGACTATAAAGATACCAGGCGT
TTCCCCCTGGAAGCTCCCTCGTGCGCTCTCCTGTTCCGACCCTGCCGCTTACCGGATACCTGTCCGCCT
TTCTCCCTTCGGGAAGCGTGGCGCTTTCTCATAGCTCACGCTGTAGGTATCTCAGTTCGGTGTAGGTCG
TTCGCTCCAAGCTGGGCTGTGTGCACGAACCCCCCGTTCAGCCCGACCGCTGCGCCTTATCCGGTAACT
ATCGTCTTGAGTCCAACCCGGTAAGACACGACTTATCGCCACTGGCAGCAGCCACTGGTAACAGGATTA
GCAGAGCGAGGTATGTAGGCGGTGCTACAGAGTTCTTGAAGTGGTGGCCTAACTACGGCTACACTAGAA
GGACAGTATTTGGTATCTGCGCTCTGCTGAAGCCAGTTACCTTCGGAAAAAGAGTTGGTAGCTCTTGAT
CCGGCAAACAAACCACCGCTGGTAGCGGTGGTTTTTTTGTTTGCAAGCAGCAGATTACGCGCAGAAAAA
AAGGATCTCAAGAAGATCCTTTGATCTTTTCTACGGGGTCTGACGCTCAGTGGAACGAAAACTCACGTT
AAGGGATTTTGGTCATGAGATTATCAAAAAGGATCTTCACCTAGATCCTTTTAAATTAAAAATGAAGTT
TTAAATCAATCTAAAGTATATATGAGTAAACTTGGTCTGACAGTTACCAATGCTTAATCAGTGAGGCAC
CTATCTCAGCGATCTGTCTATTTCGTTCATCCATAGTTGCCTGACTCCCCGTCGTGTAGATAACTACGA
TACGGGAGGGCTTACCATCTGGCCCCAGTGCTGCAATGATACCGCGAGACCCACGCTCACCGGCTCCAG
ATTTATCAGCAATAAACCAGCCAGCCGGAAGGGCCGAGCGCAGAAGTGGTCCTGCAACTTTATCCGCCT
CCATCCAGTCTATTAATTGTTGCCGGGAAGCTAGAGTAAGTAGTTCGCCAGTTAATAGTTTGCGCAACG
TTGTTGCCATTGCTACAGGCATCGTGGTGTCACGCTCGTCGTTTGGTATGGCTTCATTCAGCTCCGGTT
CCCAACGATCAAGGCGAGTTACATGATCCCCCATGTTGTGCAAAAAAGCGGTTAGCTCCTTCGGTCCTC
CGATCGTTGTCAGAAGTAAGTTGGCCGCAGTGTTATCACTCATGGTTATGGCAGCACTGCATAATTCTC
TTACTGTCATGCCATCCGTAAGATGCTTTTCTGTGACTGGTGAGTACTCAACCAAGTCATTCTGAGAAT
AGTGTATGCGGCGACCGAGTTGCTCTTGCCCGGCGTCAATACGGGATAATACCGCGCCACATAGCAGAA
CTTTAAAAGTGCTCATCATTGGAAAACGTTCTTCGGGGCGAAAACTCTCAAGGATCTTACCGCTGTTGA
GATCCAGTTCGATGTAACCCACTCGTGCACCCAACTGATCTTCAGCATCTTTTACTTTCACCAGCGTTT
CTGGGTGAGCAAAAACAGGAAGGCAAAATGCCGCAAAAAAGGGAATAAGGGCGACACGGAAATGTTGAA
TACTCATACTCTTCCTTTTTCAATATTATTGAAGCATTTATCAGGGTTATTGTCTCATGAGCGGATACA
TATTTGAATGTATTTAGAAAAATAAACAAATAGGGGTTCCGCGCACATTTCCCCGAAAAGTGCCAC

DNA sequence of pBNMB-HA-cre1

GTGGCACTTTTCGGGGAAATGTGCGCGGAACCCCTATTTGTTTATTTTTCTAAATACATTCAAATATGT
ATCCGCTCATGAGACAATAACCCTGATAAATGCTTCAATAATATTGAAAAAGGAAGAGTATGAGTATTC
AACATTTCCGTGTCGCCCTTATTCCCTTTTTTGCGGCATTTTGCCTTCCTGTTTTTGCTCACCCAGAAA
CGCTGGTGAAAGTAAAAGATGCTGAAGATCAGTTGGGTGCACGAGTGGGTTACATCGAACTGGATCTCA
ACAGCGGTAAGATCCTTGAGAGTTTTCGCCCCGAAGAACGTTTTCCAATGATGAGCACTTTTAAAGTTC
TGCTATGTGGCGCGGTATTATCCCGTATTGACGCCGGGCAAGAGCAACTCGGTCGCCGCATACACTATT
CTCAGAATGACTTGGTTGAGTACTCACCAGTCACAGAAAAGCATCTTACGGATGGCATGACAGTAAGAG
AATTATGCAGTGCTGCCATAACCATGAGTGATAACACTGCGGCCAACTTACTTCTGACAACGATCGGAG
GACCGAAGGAGCTAACCGCTTTTTTGCACAACATGGGGGATCATGTAACTCGCCTTGATCGTTGGGAAC
CGGAGCTGAATGAAGCCATACCAAACGACGAGCGTGACACCACGATGCCTGTAGCAATGGCAACAACGT

TGCGCAAACTATTAACTGGCGAACTACTTACTCTAGCTTCCCGGCAACAATTAATAGACTGGATGGAGG
CGGATAAAGTTGCAGGACCACTTCTGCGCTCGGCCCTTCCGGCTGGCTGGTTTATTGCTGATAAATCTG
GAGCCGGTGAGCGTGGGTCTCGCGGTATCATTGCAGCACTGGGGCCAGATGGTAAGCCCTCCCGTATCG
TAGTTATCTACACGACGGGGAGTCAGGCAACTATGGATGAACGAAATAGACAGATCGCTGAGATAGGTG
CCTCACTGATTAAGCATTGGTAACTGTCAGACCAAGTTTACTCATATATACTTTAGATTGATTTAAAAC
TTCATTTTTAATTTAAAAGGATCTAGGTGAAGATCCTTTTTGATAATCTCATGACCAAAATCCCTTAAC
GTGAGTTTTCGTTCCACTGAGCGTCAGACCCCGTAGAAAAGATCAAAGGATCTTCTTGAGATCCTTTTT
TTCTGCGCGTAATCTGCTGCTTGCAAACAAAAAAACCACCGCTACCAGCGGTGGTTTGTTTGCCGGATC
AAGAGCTACCAACTCTTTTTCCGAAGGTAACTGGCTTCAGCAGAGCGCAGATACCAAATACTGTCCTTC
TAGTGTAGCCGTAGTTAGGCCACCACTTCAAGAACTCTGTAGCACCGCCTACATACCTCGCTCTGCTAA
TCCTGTTACCAGTGGCTGCTGCCAGTGGCGATAAGTCGTGTCTTACCGGGTTGGACTCAAGACGATAGT
TACCGGATAAGGCGCAGCGGTCGGGCTGAACGGGGGGTTCGTGCACACAGCCCAGCTTGGAGCGAACGA
CCTACACCGAACTGAGATACCTACAGCGTGAGCTATGAGAAAGCGCCACGCTTCCCGAAGGGAGAAAGG
CGGACAGGTATCCGGTAAGCGGCAGGGTCGGAACAGGAGAGCGCACGAGGGAGCTTCCAGGGGGAAACG
CCTGGTATCTTTATAGTCCTGTCGGGTTTCGCCACCTCTGACTTGAGCGTCGATTTTTGTGATGCTCGT
CAGGGGGGCGGAGCCTATGGAAAAACGCCAGCAACGCGGCCTTTTTACGGTTCCTGGCCTTTTGCTGGC
CTTTTGCTCACATGTTCTTTCCTGCGTTATCCCCTGATTCTGTGGATAACCGTATTACCGCCTTTGAGT
GAGCTGATACCGCTCGCCGCAGCCGAACGACCGAGCGCAGCGAGTCAGTGAGCGAGGAAGCGGAAGAGC
GCCCAATACGCAAACCGCCTCTCCCCGCGCGTTGGCCGATTCATTAATGCAGCTGGCACGACAGGTTTC
CCGACTGGAAAGCGGGCAGTGAGCGCAACGCAATTAATGTGAGTTAGCTCACTCATTAGGCACCCCAGG
CTTTACACTTTATGCTTCCGGCTCGTATGTTGTGTGGAATTGTGAGCGGATAACAATTTCACACAGGAA
ACAGCTATGACCATGATTACGCCAAGCGCGCAATTAACCCTCACTAAAGGGAACAAAAGCTGGAGCTCC
ACCGCGGTGGCGGCCGCCTCGAGTCTAGAGTTGTTTGGATAATTAGATCTCTCTCTTTCTATCGTATTT
TGCAATAATAGGTATTAACTTTTATACTGATTGTTAGTAGATGCCTTCAAATTTCTTTTTATTTAAAT
TCATATGCTATATCTTTTAAAACACTCCACATTTTATTGTTGCTAACTGTGCTATTGATCTTTAAGTCA
ATAGCTGCTCATTTTGTTGAACTCCACAGAGACACTAAATTTGTTTATTTTGATGGATGCTTTATAATT
AAAGTTACGTAATCTGCTTGACATTTAGCCAACTATATAAAAAAGATCAAAATGTAGCTTAAATCTCAA
AAAATCATCATAATTTACTATCAAATTATTAAGAAATTCATATAATCACCACTTTATTGACTTTTATTC
ATCTTATAGAGTGATAGTAGAGTTGAGCCAAATTGATACTTGTTTACGTTGTATTATTTTGAAATTTTA
AAAAAATGAAAATGAGAGAAAAATTTATTTAAATTTGAGCTTAGAATCTTTAAGGAAGATCAAAAATGG
GCTAACTAAATGTTAGAGTACGAAGACTGTTCTTGAAATAAAGTGTCATCCTTTACGAATCAAGTTGCT
ACTTTAATGAATAATAGAATTTGAGGTAGAGCTAAAATGAGAGATATAGTAATGCTATTGGATTATATT
TGGTTTGTATGATGGTTTTTCTTTGGTAAATGAATGATATAAATGAAGAGTGGCAATAAAATTAATTGA
AATTGAATGAAAAAATGAATAGAAATTAAAGAAGAGTATAATTTTATTTTTGAATTTTATTTAAATTTT
AATGCGTGTATTTATTTGGGTGATGTCGACGGTATCGATAAGCTTGATATCTTCAAAGTATGGATTAAT
TATTTCAAATTATTAGAAGGTAATTAATCTGCATAAATTCAAAACTATAAAAATAAAACATTAAAATTA
ATTCAACCTTATTGAAGCATCAAAATCTGAATCTCTAGAAAGACTGATTCTGATTGGATAATTTTTCGG
CGCTAAGGATTTTGGATTAAAGAAAATTAGATTTAATTATTAATCATGATTTGAATAGGATAGCAAGAA
TATTTGTTTGGTTTAAAAGGGAAAGCGGGTAATTATCAAAAATTTATAAATAATTTTAAAACAATAAAT
AGAAAAACAAATAAGATTATAAAAACTTACAAAAATGATTGAACAAGATGGTTTACACGCTGGTTCTCC
CGCCGCTTGGGTCGAAAGACTTTTCGGTTATGACTGGGCTCAACAAACCATCGGTTGCTCTGATGCCGC
CGTCTTCCGTCTTTCTGCTCAAGGTCGTCCTGTTCTTTTCGTCAAGACCGACCTTTCTGGTGCCCTTAA
TGAACTTCAAGATGAAGCTGCCCGTCTTTCTTGGCTTGCCACCACCGGTGTTCCTTGCGCTGCTGTCCT
TGACGTTGTCACTGAAGCCGGTAGAGACTGGCTTCTTTTAGGTGAAGTCCCCGGTCAAGATCTTCTTTC
TTCTCACCTTGCTCCTGCCGAAAAAGTTTCTATCATGGCTGATGCTATGCGTCGTCTTCATACCCTTGA
TCCCGCTACCTGCCCTTTCGACCACCAAGCCAAACATCGTATCGAACGTGCTCGTACTCGTATGGAAGC
CGGTCTTGTCGATCAAGATGATCTTGACGAAGAACATCAAGGTCTTGCCCCTGCCGAACTTTTCGCCAG
ACTTAAGGCCCGTATGCCCGACGGTGAAGATCTTGTCGTCACCCATGGTGATGCCTGCTTACCCAATAT
CATGGTTGAAAATGGTCGTTTTTCTGGTTTCATCGACTGTGGTCGTCTTGGTGTCGCCGACCGTTATCA
AGATATTGCCTTAGCTACCCGTGATATTGCTGAAGAACTTGGTGGTGAATGGGCTGACCGTTTCCTTGT
CCTTTACGGTATCGCCGCTCCCGATTCTCAACGTATCGCCTTCTATCGTCTTCTTGACGAATTCTTCTG
AGATCCTTAAATTAAAAATTCAATATATATTTACAAACTTTCATATAAAATAAATATATTATATAAAAT
TAATTTTTAGTGTATTATATTAACATTAAAGCACCAAAAAAACGTGTTAATATACTACTATAAAATATA
ATTTATTCCAAATTGACTAAAATCATTATTTTACAACTCATTTGTATATATATTTTATGTCAATTATTT
TTTTTAACTTTCTAAAAAAAAAAATTCCTCTTCACATACATGTTAGCTCTTAAAAATTTGTCTGCAAAT
CCAATAATAATATTTTTTTTTGCCATTAAATTTTCAAATTTTTACTGGAAAAATGCAGGGGGATCAGAC
AATTTATTTCTAAAAAATATTTAAAAATAAAAAATAATAAGGGTTTTGAATAACTCCTTTAATTTAAAT
ACACATTTTTAAATTTTTTTTAGCTCTTTAAATATTCATAAAAATAAAAAATAACTAACTAAAAATAAA
TAAAAAGATAATAATGATTAAAGGTATAATACTGAATAAGAAAAAACATAATAGAGTACTTATTTTTTA
TATCACTATTTTTAATATCTTGAAAGCAAAACTTTTTTATATATCTTAAAATATATTGTATCGTTTATT
CAATTATTTTCTTTAAATTTCAAATATATTGATAAAAAAGATGACATGTTTTTTAAAGAAAACATGAAA
TATAAAATAGATAAATATCAATTATTTTATTTATTAAATATATAAGCTGCTCAAAACATAGCTCATTCA
TCAATTATAATATGTGAATCATTAATTTTCAAAATATTACTCATTATTTAGGCTATCATTTATTTTTTA

TTTTCAATTATCCGTTTCTATTATATTTTAATATTAAGTTGTGATTCTTGAATTTTGTGTCATGAATTA
TTTGTAAATCTTTTTATTTCTGATAAAAAATATAAATTGATTGACTCATGATTTAAATCATGAGTCAAC
CTAACTAATTTTCAAAATTCTTCTATTCTAAAATATAGATGTGATTCTTGAATCTCTCTTGAATATAAA
GTAATTTTTTATATTTCTGATATAATTCTTAGCTACGTGATTCACGATTTATGCAATGATCCATATAAA
ATAATGTAAATAGTGTATATATATATATTCGTCTTTTTTATTCTTTATATAATTTAAAAAAATTAAAAA
AATTTAATAAAGCTCTAATAAAATAAATAATAATACTAAACTTAAACATATGTATCCTTATGATGTTCC
TGATTATGCTGGTGCTAGCAACCTGCTGACCGTTCATCAGAATCTGCCGGCGCTGCCGGTGGATGCCAC
CAGCGATGAAGTGCGCAAAAACCTGATGGATATGTTTCGTGATCGTCAGGCCTTCAGCGAACATACCTG
GAAAATGCTGCTGAGCGTGTGCCGTAGCTGGGCCGCGTGGTGCAAACTGAACAACCGTAAATGGTTTCC
GGCGGAACCGGAAGATGTGCGTGATTATCTGCTGTATCTGCAGGCGCGTGGCCTGGCCGTGAAAACCAT
CCAGCAGCACCTGGGTCAGCTGAACATGCTGCATCGCCGTAGCGGCCTGCCGCGCCCGAGCGATAGCAA
TGCGGTGAGCCTGGTGATGCGTCGTATTCGTAAAGAAAACGTGGATGCGGGTGAACGTGCGAAACAGGC
CCTGGCGTTCGAACGCACCGATTTTGATCAGGTTCGTAGCCTGATGGAAAACAGCGATCGCTGCCAGGA
TATTCGCAACCTGGCGTTTCTGGGTATTGCGTATAATACCCTGCTGCGCATCGCCGAAATTGCGCGCAT
TCGTGTTAAAGATATTAGCCGCACCGATGGCGGTCGTATGCTGATCCACATCGGTCGCACCAAAACCCT
GGTGAGCACCGCGGGCGTGGAAAAAGCCCTGAGCCTGGGTGTGACCAAACTGGTGGAACGTTGGATTAG
CGTGAGCGGTGTTGCGGATGATCCGAACAACTATCTGTTTTGCCGTGTGCGCAAAAATGGCGTGGCGGC
GCCGAGCGCGACCAGCCAGCTGAGCACCCGTGCCCTGGAAGGCATTTTTGAAGCCACCCATCGCCTGAT
TTATGGTGCGAAAGATGATAGCGGTCAGCGTTATCTGGCCTGGAGCGGTCATAGCGCCCGCGTGGGTGC
GGCGCGCGATATGGCGCGTGCGGGTGTGAGCATCCCGGAAATTATGCAGGCCGGCGGTTGGACCAATGT
GAACATTGTGATGAACTATATTCGCACCCTGGATAGCGAAACCGGTGCGATGGTGCGCCTGCTGGAAGA
TGGCGATTGACGACGCGTGATGATGATCCAGATCTACTAGTTGAGCGAACTGAATCGGTCAGCTAAACC
AACCAATCAACATAATAAACTTTATTATTTTTACTTAAGCATCTTACTGTTGTTGTAATAGTAGAGAAA
GAAATACCCAATTAACTTCATTCACATAACATTAATATCTATAAACATCTTTTTTCTCACATATATACA
ACTCTCTAAATCAACAAATAACTTTTTAAAAATAATGGATATATATTAACAAAATAATATATCTCTTTT
TACAAAATAGTTCTTATATAAATACGTATTCTGCACTCACCCGCATTTTTCACAACAAAAACATACCAA
AAAAATTCTTACTTCTACATGTTTCCTTTCTTATTATTACAAAATTATTTTATAAATAGCATACAAAAA
TAAATACAATAAAAAAATAAACAAAATCCTTTTTTATTTTGAATTATTTAAAACAAATATTTTCAATCA
ATCAGTCAGTCAGCATAATATTAAAGCAACAAAACAAACCCAAGTTGTTTTTATAGTTTTTTAATTGCT
TTTCAGTACTATAAATAAATTTGTTATTACTTCAAGATTGATAAACTTCTTTTTTAAATTAAATATCTA
TGAATGAATAAATAAGTTGATATCTCTTTTTAACTTGTTTTCCTCTCTTTTACTTACTTGCCAATTTTT
TTTTTAAATTAAAGAAATATCTTTTTATTTTTCAAAAACAAAATTTATTTTCCCTTGTATACAAAAACC
CCTTTATTTAAATAAAATCTTTATGCCCATCAATAGCCACATCTTCTCGAGGGGGGGCCCGGTACCCAA
TTCGCCCTATAGTGAGTCGTATTACGCGCGCTCACTGGCCGTCGTTTTACAACGTCGTGACTGGGAAAA
CCCTGGCGTTACCCAACTTAATCGCCTTGCAGCACATCCCCCTTTCGCCAGCTGGCGTAATAGCGAAGA
GGCCCGCACCGATCGCCCTTCCCAACAGTTGCGCAGCCTGAATGGCGAATGGACGCGCCCTGTAGCGG
CGCATTAAGCGCGGCGGGTGTGGTGGTTACGCGCAGCGTGACCGCTACACTTGCCAGCGCCCTAGCGCC
CGCTCCTTTCGCTTTCTTCCCTTCCTTTCTCGCCACGTTCGCCGGCTTTCCCCGTCAAGCTCTAAATCG
GGGGCTCCCTTTAGGGTTCCGATTTAGTGCTTTACGGCACCTCGACCCCAAAAAACTTGATTAGGGTGA
TGGTTCACGTAGTGGGCCATCGCCCTGATAGACGGTTTTTCGCCCTTTGACGTTGGAGTCCACGTTCTT
TAATAGTGGACTCTTGTTCCAAACTGGAACAACACTCAACCCTATCTCGGTCTATTCTTTTGATTTATA
AGGGATTTTGCCGATTTCGGCCTATTGGTTAAAAAATGAGCTGATTTAACAAAAATTTAACGCGAATTT
TAACAAAATATTAACGCTTACAATTTAG
